# Advances in nonlinear metasurfaces for imaging, quantum, and sensing applications

**DOI:** 10.1515/nanoph-2023-0526

**Published:** 2023-11-21

**Authors:** Ze Zheng, Davide Rocco, Hang Ren, Olga Sergaeva, Yipei Zhang, K. Birgitta Whaley, Cuifeng Ying, Domenico de Ceglia, Constantino De-Angelis, Mohsen Rahmani, Lei Xu

**Affiliations:** Department of Engineering, Advanced Optics and Photonics Laboratory, School of Science Technology, Nottingham Trent University, Nottingham, UK; Department of Information Engineering, University of Brescia, Brescia, Italy; Department of Chemistry, University of California, Berkeley, CA, USA

**Keywords:** nonlinear metasurfaces, nonlinear imaging, spontaneous parametric down-conversion, sensing

## Abstract

Metasurfaces, composed of artificial meta-atoms of subwavelength size, can support strong light–matter interaction based on multipolar resonances and plasmonics, hence offering the great capability of empowering nonlinear generation. Recently, owing to their ability to manipulate the amplitude and phase of the nonlinear emission in the subwavelength scale, metasurfaces have been recognized as ultra-compact, flat optical components for a vast range of applications, including nonlinear imaging, quantum light sources, and ultrasensitive sensing. This review focuses on the recent progress on nonlinear metasurfaces for those applications. The principles and advances of metasurfaces-based techniques for image generation, including image encoding, holography, and metalens, are investigated and presented. Additionally, the overview and development of spontaneous photon pair generation from metasurfaces are demonstrated and discussed, focusing on the aspects of photon pair generation rate and entanglement of photon pairs. The recent blossoming of the nonlinear metasurfaces field has triggered growing interest to explore its ability to efficiently up-convert infrared images of arbitrary objects to visible images and achieve spontaneous parametric down-conversion. This recently emerged direction holds promising potential for the next-generation technology in night-vision, quantum computing, and biosensing fields.

## 1 Introduction

Since the first observation in 1961 [1], the nonlinear generation, also called frequency conversions, has been intensively studied for nearly six decades, leading to a vast range of related applications, including nonlinear microscopy [2–4], quantum light sources [5–8], and ultrasensitive sensing [9–11]. To date, the platform carrying on the nonlinear generation has been updated from nonlinear crystals to more compact and miniature elements, such as fibers and waveguides [12–15]. Nevertheless, the basic concept of efficiently converting the fundamental light from one frequency to a new frequency still relies on utilizing the long distance along the propagating direction, where the phase-matching and propagating loss terms significantly limit its applications [13, 16, 17]. Although the periodically poled crystals have improved the dependence of conversion efficiency on phase-matching conditions, these limitations continue to affect their practical applications [18–20]. Based on these reasons, it is notable to explore a new concept as an alternative option to strongly enhance the nonlinear generation, thereby overcoming the current shortcomings.

Thanks to the recent developments of the technology for the growth and nanofabrication of nonlinear materials, metasurfaces, composed of subwavelength constituent resonant atoms, have become an ideal nanoscale platform to shape light with extremely confined and enhanced electric field [21, 22]. The abilities of metasurfaces on light confinement can be precisely controlled by the careful design of geometric parameters for each unit composing the whole metasurfaces [23–25]. Currently, many works have reported on the utilization of metasurfaces to control the intensity, phase change, and polarization of incident electromagnetic waves, covering from deep UV to microwaves [21–23]. This kind of control can be independently implemented in each pixel of metasurfaces, offering massive flexibility and compatibility for real-world applications. To date, the control of light properties using metasurfaces has been dilated into the freedom of frequency by employing nonlinear generation.

Instead of relying on the propagating distance of light, nonlinear metasurfaces can support highly-concentrated light confinement based on the excitation of multipolar resonances and plasmonics. This unique approach can significantly boost nonlinear generation [26–41]. The conversion efficiency of nonlinear metasurfaces can be several hundred times higher compared to the same size of unpatterned films [42, 43]. Nowadays, many works reported on enabling enhanced nonlinear generation by nonlinear metasurfaces, including second-harmonic generation (SHG), third-harmonic generation (THG), sum-frequency generation (SFG), four-wave mixing (FWM), and high-order harmonic generation [44–53]. Nonlinear materials used in metasurfaces vary from metallic materials to second-order and third-order dielectric materials and semiconductors, such as gold, LiNbO_3_, AlGaAs, GaP, ZnO, and silicon [45, 51, 54], [55], [56]. Due to the subwavelength thickness, the light confinement and the light–matter interaction within metasurfaces play a crucial role in improving the conversion efficiency of nonlinear generation, instead of being limited by propagation losses and phase-matching condition.

Bound states in the continuum (BICs), originating from quantum mechanics, have now been admitted as an ideal tool to manipulate the light confinement within metasurfaces [57]. The concept of BICs has been extensively observed and applied in constructing nonlinear metasurfaces and enhancing the nonlinear generation [47, 52, 55, 58, 59]. By breaking the symmetry, BICs can be transformed into high-Q resonances, possessing controllable light confinement within metasurfaces, and hence tremendously enhancing the nonlinear generation. Moreover, nonlinear metasurfaces have shown remarkable potential in the wavefront shaping and phase control of harmonic waves [60]. These properties of nonlinear metasurfaces further expand their applications, one of which is nonlinear imaging.

The nonlinear imaging technique utilizes nonlinear generation to create and convert images, thereby surpassing the limitations of linear optics, which is limited by the incident light frequencies [61]. Nonlinear imaging has attracted increasing attention due to its extensive potential in future applications, including sensing, night vision, and spectroscopy [61, 62]. Nonlinear metasurfaces, serving as exceptional imaging components, offer unique compatibility and flexibility for application in nonlinear imaging. Benefiting from the enhanced nonlinear generation, nonlinear metasurfaces can efficiently convert the infrared image into visible light, realizing the concepts of night vision and infrared imaging [61–63]. Due to the eased phase-matching condition, nonlinear imaging techniques based on metasurfaces have a larger acceptance angle and a wider operating range compared to other platforms, such as bulk crystals, fibers, and waveguides. Additionally, with the ability of wavefront engineering, nonlinear metasurfaces bring futuristic possibilities into current nonlinear imaging techniques, such as nonlinear holography [64–66] and nonlinear metalenses [60, 67, 68]. Via encoding the amplitude and phase distribution on each subwavelength pixel of metasurface, the concealed image becomes discernible solely within the nonlinear signal. This concept is known as image encryption. Under the required phase distribution, nonlinear metasurfaces can extend their functions, serving as compact lenses. These metalenses lead to the development of multifunctional nonlinear metasurfaces, crucially broadening their practical use.

Over the last decades, quantum optics played an increasingly important role in quantum computing as one of the fundamental ways of constructing and building quantum systems [69]. To date, the photon-based quantum computer specialized in the boson sampling problem has been realized in 2020 [70]. However, it is rather difficult to build up such a complex optical system with numerous bulky components for commercial applications. Aiming for manipulating and controlling the photons at the nanoscale, the concept of integrated photonic quantum computing attracts tremendous attention to replace traditional bulky optical blocks [71, 72]. Metasurfaces, with extraordinary capabilities of tailoring and manipulating light at the nano-scale, have been recognized as a feasible platform to be applied in a vast range of quantum optical technologies, including quantum light sources [73–75], photon operator [76, 77], and quantum tomography [78]. Particularly, nonlinear metasurfaces have been widely applied in constructing quantum light sources to generate photon pairs with high efficiency and entanglement in the momentum and polarization domain [74, 79]. It has been generally revealed as a promising approach for the generation of entangled photon pairs via carefully designing the resonant response of nonlinear metasurfaces.

Optical sensors, detecting absorption, emission, scattering, dielectric constant, and chirality of biological samples, have an increasingly important role in the biosensing field. Compared to linear optical interaction, nonlinear generation enables high-sensitivity sensing at low concentrations. As super-compact and feasible nanodevices, nonlinear metasurfaces facilitate the detection of nonlinear light–matter interaction within biological samples, broadening the accessibility of nonlinear biosensing or bioimaging for a wider range of applications [68, 80, 81].

**Figure 1:**
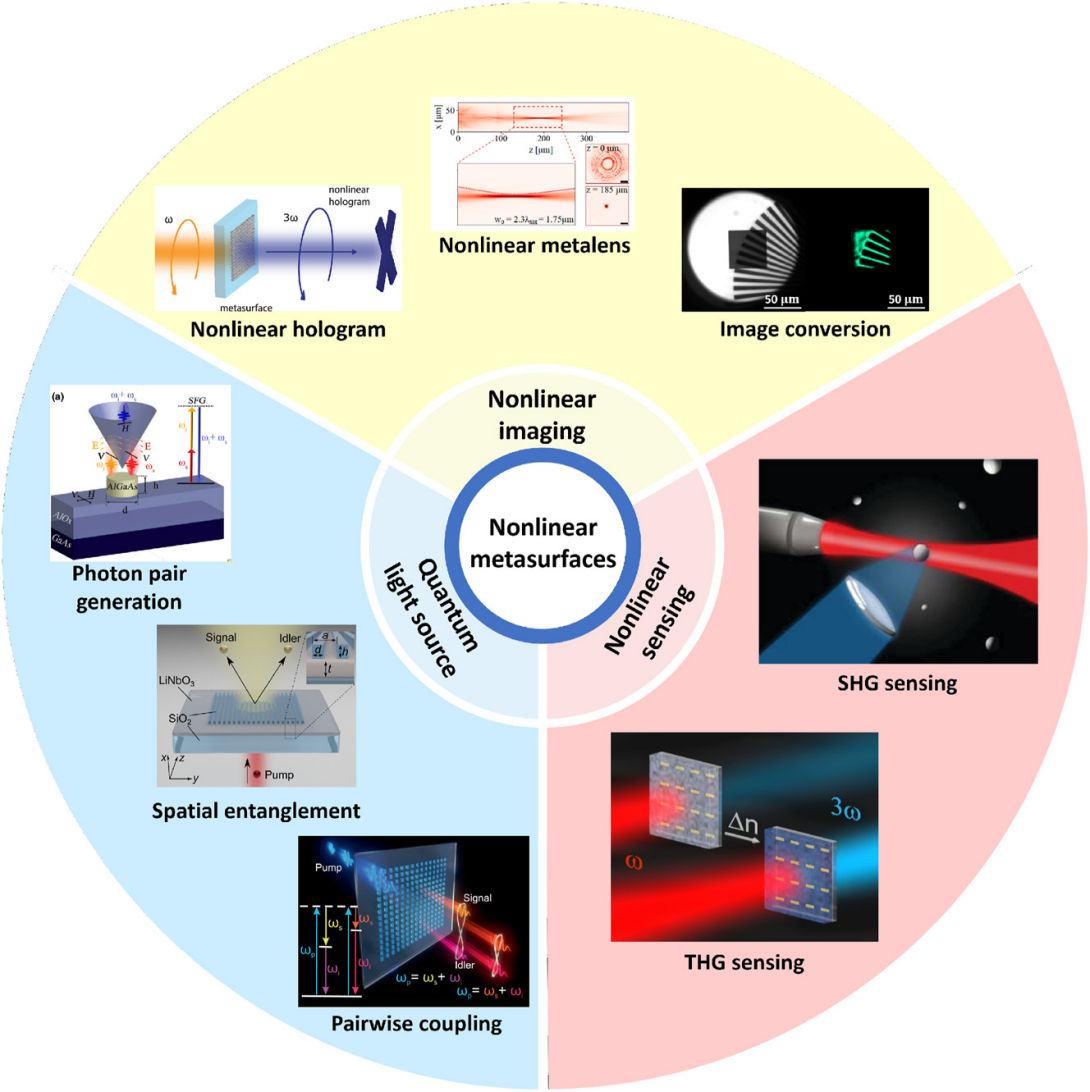
Overview of the diverse applications of nonlinear metasurfaces. Nonlinear imaging part: the figures from left to right are respectively from [62, 64, 82]. Quantum light source part: the figures from top to bottom are respectively from [73–75]. Nonlinear sensing part: the figures from top to bottom are respectively from [9, 80] with permission (Copyright 2012 and 2016, American Chemical Society).

Considering the rapid growth of the nonlinear metasurfaces research field, there are several review papers focusing on the development of nonlinear metasurfaces instead of their related applications [26–30, 32]. Here we introduce the evolution of nonlinear metasurfaces and their close connections with other fields, including nonlinear imaging, quantum computing, and nonlinear sensing (Figure 1). This paper is organized as demonstrated below. Section 2 provides an overview of nonlinear metasurfaces and outlines several approaches to enhance nonlinear generation. In Section 3, we discuss the use of nonlinear metasurfaces for nonlinear holography, nonlinear metalenses, and image conversion. In Section 4, we review the increasing role of nonlinear metasurfaces in constructing quantum light sources. In the last section, we provide an outlook on developments of metasurfaces in the quantum computing and nonlinear sensing fields.

## 2 Nonlinear optical interaction in metasurfaces

In this section, we briefly review the current development on enhancing nonlinear light–matter interaction with metasurfaces, and showcase several examples that employ different approaches to enhance optical nonlinear generation and facilitate the realization of nonlinear metasurfaces. These cutting-edge approaches have greatly expanded the ability to harness and control nonlinear processes, opening up promising avenues for further advancements in nonlinear applications.

Nonlinear optical processes are universally described by the Taylor expansion of the polarization *P*(*t*) depending on the electric field strength (which must be large) *E*(*t*) [16, 83]:

(1) $$P\left(t\right)=\epsilon_{0}\left(\chi^{\left(1\right)}E_{0}\left(t\right)+\chi^{\left(2\right)}E_{0}^{2}\left(t\right)+\chi^{\left(3\right)}E_{0}^{3}\left(t\right)+\cdots \;\right)$$

where *ϵ*_0_ – is the dielectric permittivity of vacuum, and *χ* ^(*x*)^ stands for the dielectric susceptibility tensor of *x*th order related to the symmetry properties of the crystal lattice. The first order, *χ* ^(1)^, describes the linear response while the second – *χ* ^(2)^ – (being the first nonlinear term) is responsible for the second harmonic generation (SHG), as well as sum-frequency generation (SFG), spontaneous parametric down-conversion (SPDC) and difference-frequency generation (DFG) [84] in nonlinear materials with no inversion symmetry of the crystal lattice (this applies to all even-order processes as all susceptibilities of even orders are equal to zero in isotropic and centrosymmetric materials). The term *χ* ^(3)^ defines instead the third order nonlinear response including third harmonic generation (THG) and four-wave mixing (FWM), Raman effect, Kerr effect, which can manifest in symmetrical materials. Please note that further terms in eq. (1) are involved in higher-order harmonic generation. The *χ* ^(2)^ is a third-rank tensor and consists of 27 elements, and *χ* ^(3)^ is a fourth-rank tensor with 81 separate elements, but some of them are equal to zero and some others are not independent due to the symmetry of the crystal lattice, which can be deduced based on the type of the crystal lattice (cubic, hexagonal, etc.) [16]. Let us underline that the phase matching condition between involved waves must be satisfied in conventional nonlinear processes in bulk materials, but it becomes negligible in nanostructures and metasurfaces. Nanostructured materials also overcome the limitation on the manifestation of second-order nonlinear processes in centrosymmetric materials by breaking the symmetry at the surface of the nanostructure or the interface between different materials [85–91] and using various types of optical resonances, including plasmon and Mie resonances, guided-mode resonances, and BICs [26–41, 92–94].

**Figure 2:**
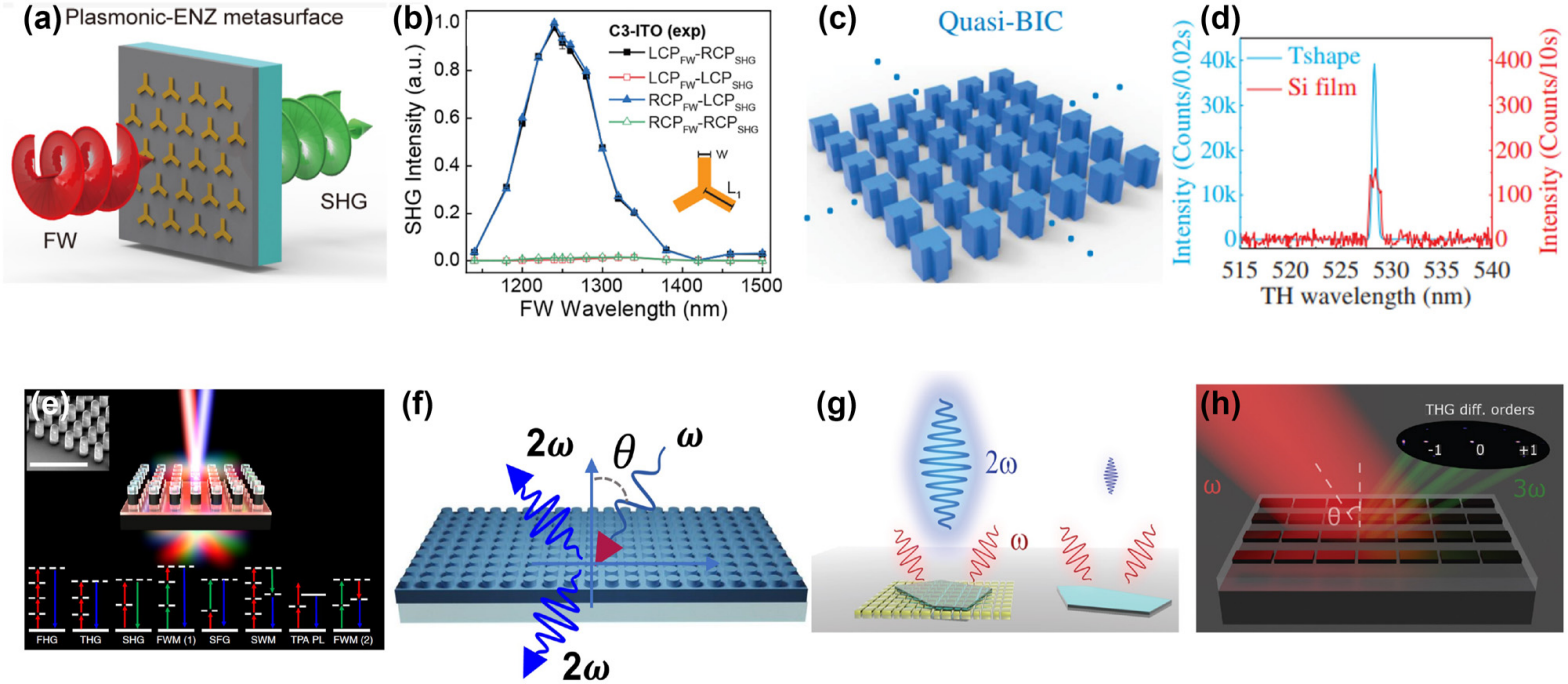
Optical nonlinear processes with resonant metasurfaces. (a) Schematic illustration of SHG with plasmonic-ENZ metasurface consisting of Au meta-atoms on an indium-tin-oxide (ITO) layer. Adapted from [130]. Copyright 2020, American Chemical Society. (b) Experimentally measured polarization-dependent SHG spectra of hybrid plasmonic-ENZ C_3_-ITO metasurface. Adapted from [130]. Copyright 2020, American Chemical Society. (c) Si metasurface with symmetric defects that supports quasi-BICs. Adapted from [138]. (d) comparison of the THG in quasi-BIC metasurface and a reference Si thin film with the same thickness as the metasurface. Adapted from [138]. (e) Schematic illustration of an optical metamixer consisting of a square array of subwavelength GaAs dielectric resonators, where seven nonlinear optical processes occur simultaneously under two femtosecond near-IR pulses pump. Adapted from [152]. (f) Schematic illustration of boosting SHG from a metasurface-slab hybrid system driven by high-Q guided resonances and bound states in the continuum [153]. (g) Illustration of enhanced SHG from TMDs combined with a Si metasurface hosting BICs. Adapted from [58]. Copyright 2020, American Chemical Society. (h) Illustration of THG diffraction tailoring by hybrid modes in Si high-Q metasurfaces. Adapted from [49]. Copyright 2021, American Chemical Society.

In the process of SHG, which was the first discovered nonlinear generation process (1961 [1]), the electron in the material absorbs two incident photons with frequency *ω* and emits one photon at a double frequency 2*ω* (Figure 2(g)) [1, 95, 96]. In 1961, the theoretical description of various nonlinear generation effects (SHG, SFG, THG, FWM) was published [95]. SFG was also first experimentally shown in 1962 [97]; in this nonlinear process two laser photons with two wavelengths being absorbed in the media induce the emission of a photon with a third wavelength, according to the energy conservation law, in the frequency terms it can be written as *ω*_1_ + *ω*_2_ = *ω*_3_ (Figure 6(a), left panel). Please note that the SHG can be considered a case of SFG with *ω*_1_ = *ω*_2_ = *ω*. The conservation of momentum requires the wave vectors to obey the phase-matching condition *k*_3_ = *k*_1_ + *k*_2_ + Δ*k* with |Δ*k*| ≪ |*k*_3_|, which can be difficult to satisfy because of the material dispersion [16, 83, 95]. Polarization in SFG is written as *P*(*ω*_1_ + *ω*_2_) = 2*ϵ*_0_ *χ* ^(2)^ *E*_0_(*ω*_1_)*E*_0_(*ω*_2_). In the process of DFG (SPDC) *ω*_3_ = *ω*_1_ − *ω*_2_ and $P\left(\omega_{1}-\omega_{2}\right)=2\epsilon_{0}\chi^{\left(2\right)}E_{0}\left(\omega_{1}\right)E_{0}^{*}\left(\omega_{2}\right)$ [16]. In terms of optical parametric generation SPDC (Figure 6(a), right panel, and (b))) is a parametric process of the spontaneous decay of absorbed pump photon with frequency *ω*_1_ into a pair of output signal (*ω*_3_) and unwanted idler (*ω*_2_) photons via two-photon emission [16]. In the parametric processes, virtual short-living electron energy levels are involved, while in the non-parametric processes, like Raman scattering, the initial and final states are the real electron energy levels, and the photon energy is partially irretrievably absorbed. Theoretical description of optical parametric processes was published in 1961 [98] and the first quantum mechanical description of SPDC was published in 1967 [99], later the experimental demonstration was published [100–102], and ideas of using parametric processes for image conversion and holography started to emerge [103–105]. THG was demonstrated in [106]. THG can be considered a case of FWM with *ω*_1_ = *ω*_2_ = *ω*_3_; FWM in a non-degenerate case is a process that originates from the interaction of three incident waves in a medium characterized by the susceptibility *χ* ^(3)^ generating the fourth one *ω*_4_ = *ω*_1_ + *ω*_2_ + *ω*_3_ [16, 95].

In nonlinear nanophotonics the characteristics of the transmitted or reflected light strongly depend on the pump polarization and direction, geometry, symmetry and orientation of the meta-atoms as well as on the crystalline structure of the material they are made of. Works on the effects of crystal symmetry in harmonics generation processes started in [95, 107–110]. The interplay between the symmetry of a nanoparticle and the crystal structure of its material and the polarization of the pump and the generated signal in nonlinear generation processes was discussed in [31, 111–124], etc. for plasmonic and semiconductor centrosymmetric and non-centrosymmetric materials.

Plasmonic and dielectric nanostructures have been extensively investigated to create various shapes and clusters to enhance the optical nonlinear generation through the excitation of plasmonic resonances or multipolar Mie-type resonances. By designing the localized surface plasmon resonances at the excitation or harmonic emission wavelengths, nonlinear harmonic generation can be significantly enhanced using plasmonic nanoantennas [125]. The strong local field enhancement near the nanoantenna’s surface can induce a much stronger nonlinear response as compared to traditional plasmonic materials. Substantial efforts have been devoted to advancing nonlinear generation using plasmonic metasurfaces, often in combination with various designing approaches. For instance, researchers have explored the coupling of electromagnetic modes supported by plasmonic metasurfaces with quantum-engineered electronic intersubband transitions in semiconductor heterostructures. This experimental work [126] has demonstrated the possibility of engineering virtually any element of the nonlinear susceptibility tensor in these structures. As a notable achievement, a 400-nm-thick metasurface with a nonlinear susceptibility exceeding 5 × 10^−4^ pm/V for SHG at an approximately 8-μm wavelength under normal incidence has been realized. Various other combinations have also been investigated in this context. For instance, materials with epsilon near zero (ENZ) properties, such as indium tin oxide (ITO), have shown strong nonlinearity and the ability to confine light, reducing the requirement for phase matching conditions [127–129]. However, in ITO material, the anisotropy of the nonlinear response and the continuity of the electric field components normal to the interface typically result in a general enhancement of the nonlinear signal only under the oblique incidence of the pump light on the ITO films. To address this limitation, researchers have designed a hybrid metasurface comprising plasmonic nanostructures on an ENZ nanofilm: A hybrid plasmonic-ENZ metasurface was constructed using 30-nm thick plasmonic (Au) meta-atoms with 3-fold (C_3_) rotational symmetry. These meta-atoms were arranged in a hexagonal lattice with a period of 550 nm and then positioned on an ITO layer. The metasurface was composed of a 30 nm Au layer, followed by a 15 nm ITO layer, and a SiO_2_ substrate [130]. Through near-field coupling between the ENZ layer and the meta-atoms on the interface, a remarkable enhancement of SHG at normal incidence was experimentally observed, enabling enhanced SHG from ITO film even when the pump light impinged perpendicularly to the surface, as illustrated in Figure 2(a and b).

The combination of plasmonic resonances and advanced engineering approaches opens up new avenues for tailoring and optimizing nonlinear optical processes in nanoscale systems. However, plasmonic materials are usually associated with significant losses at the optical frequencies, resulting in relatively low damage thresholds. Moreover, the field is localized near the interface of plasmonic nanostructures, leading to a relatively small mode volume. All of these limitations have hindered the practical applications of nonlinear plasmonic metasurfaces. In contrast, nonlinear dielectric metasurfaces have garnered intense exploration in recent years [26, 27, 36, 93, 131]. Made of dielectric, they exhibit exceptionally low losses at optical frequencies and can support various electric and magnetic resonant multipoles. By exciting these resonant multipoles and their interferences, strong near-field enhancement can be achieved at the pump frequency. Additionally, the nonlinear radiation can be engineered through the nonlinearly generated multipoles. The use of nonlinear dielectric metasurfaces presents a promising platform for advancing nonlinear nanophotonics towards practical applications.

Metasurfaces, due to the coupling between adjacent nanostructures, can generally support modes with much higher Q-factors compared to individual nanoresonators. This property plays a critical role in enhancing nonlinear light–matter interaction. A considerable amount of research has been devoted to creating high-Q resonances in metasurfaces to enhance light confinement, including conventional Mie resonances and lattice resonances, Fano resonances, topologically protected states, etc. [48, 132–137]. Modes of different symmetry classes remain completely decoupled and can form strongly bounded states with no radiation, known as symmetry-protected Bound States in the Continuum (BICs). By introducing asymmetry or defects in such systems, these BICs with infinite Q-factors can be transformed into quasi-BICs with finite Q-factors. These quasi-BICs can be externally excited under plane wave incidence, making them suitable for facilitating nonlinear light–matter interaction [50, 51, 55, 59, 138–142]. For instance, Si metasurfaces with symmetry-protected BIC modes arranged in a square lattice demonstrate a magnetic dipole type BIC at the Γ point of the first Brillouin zone [138]. By introducing an asymmetric defect in the metasurface, the Q-factors of such metasurfaces can be tuned. Researchers have experimentally achieved a Q-factor of 18,511 by engineering the symmetry properties and the number of unit cells in the metasurface. Subsequently, these high-Q quasi-BICs exhibit exceptional conversion efficiency for third harmonic generation in Si metasurfaces (Figure 2(c and d)) [138]. Materials with high nonlinearities, including second-order nonlinear processes (GaP, GaAs, etc.) and third-order nonlinear processes (Si, Ge, lithium niobate (LiNbO_3_), etc.) have been widely explored by researchers to boost the nonlinear generation in nanoresonators and metasurfaces of different shapes [48, 53, 137, 141, 143–151]. By employing multiple laser pump sources, different nonlinear processes can occur simultaneously within a single metasurface. For instance, researchers have demonstrated the generation of 11 new frequencies spanning from approximately 380 nm to 1 μm using a GaAs-based dielectric metasurface. This generation arises from 7 different concurrent nonlinear processes when pumping the metasurface with two femtosecond pulses at approximately 1.24 μm and 1.57 μm wavelength, near the magnetic and electric dipole resonances of the metasurface, creating an ultra-compact nonlinear metamixer, as illustrated in Figure 2(e) [152].

Recently, attention has been drawn to the integration of waveguides and metasurfaces to explore high-Q guided resonances through waveguide modes and Mie resonances in dielectric metasurfaces. For instance, a configuration involving a LiNbO_3_ disk array placed on a thin LiNbO_3_ film enables the transformation of guided modes supported by the LiNbO_3_ thin film into high-Q guided resonances that can be directly excited under plane-wave illumination, as illustrated in Figure 2(f). This structure can also tailor both the Q-factor and the field confinement by controlling the radiative features of the metasurface resonances. The numerical simulations predict a significant SHG efficiency of 5 % with a relatively low pump intensity of 0.4 mW/cm^2^ [153].

The metasurface platform offers the capability to manipulate both near-field confinement and far-field radiation. By integrating metasurfaces with other materials possessing strong nonlinearities, it is possible to enhance their nonlinear emission intensity as well as to control the nonlinear emission pattern. In recent years, phase-change materials have emerged as a powerful platform for nonlinear applications. These materials offer the unique capability to tune the nonlinear emission from metasurfaces, making them highly versatile for various nonlinear optical applications [154, 155]. Additionally, some of these materials exhibit large nonlinearities, enabling the formation of metasurfaces that can generate sizeable nonlinear emissions [156]. For example, placing the WS_2_ monolayer on top of the Si resonant BIC metasurface can result in a remarkable enhancement of the effective nonlinear susceptibility, as depicted in Figure 2(g). This enhancement leads to a substantial increase in SHG intensity, surpassing three orders of magnitude when compared to a WS_2_ monolayer on top of a flat Si film with the same thickness [58].

As mentioned before, nonlinear metasurfaces not only can enhance the near-field by the excitation of the resonances, but also can manipulate the nonlinear emission through engineering the multipolar excitation at the pump and harmonics [49, 145] frequencies. For example, by designing a Si metasurface supporting hybrid Mie-quasiBIC modes, the control of the TH diffraction by switching between different TH diffraction orders was achieved, as illustrated in Figure 2(h) [49]. This can be interpreted by the modification of the local electric field in the meta-atoms leading to the generation of different nonlinear multipoles and thus the switching between THG diffraction orders. The manipulation of wavefronts in the linear regime has been extensively explored through the utilisation of Huygens’ metasurfaces to achieve full 2*π* phase modulation while maintaining near-unity transmission. However, in the nonlinear regime, shaping the wavefront for nonlinear emission becomes considerably more intricate compared to the linear scenario. In this context, effective phase modulation must be accompanied by achieving high and consistent nonlinear conversion efficiency. The most used approach involves positioning the pump frequency at a carefully designed resonance to amplify the electric field and the intensity of induced nonlinear currents within each meta-atom of the metasurface. Consequently, this amplification leads to a significant enhancement in nonlinear emission. Moreover, by engineering the geometry of the meta-atoms to leverage multipolar interference effects arising from different multipoles [157–159], and by exploiting the nonlinear tensor properties of material [160], one can exert precise control over the nonlinearly generated multipoles. This control allows for the shaping of the nonlinear radiation pattern, thereby achieving both high efficiency and high directionality while enabling phase tuning. This approach presents a promising platform for a diverse range of applications, including nonlinear holography, nonlinear meta-lenses, background-free nonlinear sensing, and more.

In summary, nonlinear light–matter interaction with metasurfaces offers a versatile and powerful platform for controlling and enhancing nonlinear optical processes, with potential applications in areas such as frequency conversion, nonlinear imaging, and quantum photonics. As research in this field continues to advance, metasurfaces hold promise for transformative breakthroughs in nonlinear optics and light manipulation technologies.

## 3 Nonlinear image generation and conversion/processing based on metasurfaces

In this section, we review recent advances in nonlinear imaging enabled by metasurfaces. Indeed, nonlinear metasurfaces perform the nonlinear optical transformation incorporating many of the functionalities of their linear counterparts [67, 161], [162], [163]. Recently, research in this field has gained increasing interest thanks to many achievements in various application areas, as discussed in recent reviews on nonlinear metamaterials [26–41]. Newly, nonlinear metasurfaces have found applications in holographic multiplexing, image conversion and processing and as metalenses. In the following, we will discuss the main results concerning each of the aforementioned research streams.

### 3.1 Nonlinear metasurfaces for holography and image encoding

Holographic multiplexing based on nanoscale platforms has recently gained a lot of attention due to its potential of encapsulating different images in a small volume, hence increasing the information capacity with ultrasmall footprint [164–166]. For nonlinear holography, the image is formed at a different frequency than that of the pump (e.g., the double or triple, or sum). In [167] the authors developed a nonlinear metasurface, composed of plasmonic meta-atoms with rotational symmetry, which can hide images under fundamental frequency illumination. The hidden image can be read in SHG signal, realizing the image encryption. Employing a modified design of nonlinear metasurface composed of similar meta-atoms and illuminated by circularly polarized light, authors of [168, 169] showed information encoding in both real and Fourier space in polarization and amplitude-controlled nonlinear holography. In [170], the authors demonstrated the computer-generated hologram based on THG in the multilayer plasmonic metamaterial, which allowed generating images in transmission with low background. Subsequently, the first demonstration of THG hologram in all-dielectric metasurface was reported [171].

Nonlinear holography by metasurfaces has been demonstrated to increase the multiplexing capability by using the strong nonlinear effects due to the interaction of the incident pump field with the nonlinear medium [64, 170–173]. In fact, by appropriately designing the metasurface, it is possible to generate reconstructed images both at the linear and nonlinear frequency, which may further enhance the spectral holographic multiplexing capacity. Many pioneering works on nonlinear holography have been presented as paradigms. For instance, it has been proved that an optimized grating structure in the proximity of dielectric nanoantennas allows the phase control of the nonlinear wavefront generated by the nanoantennas [174]. In particular [174], in-plane dielectric gratings reshaped the quadrupolar SHG mode from cylindrical dielectric nanoantennas into a dipolar-like emission pattern. The SH emission profile obtained in the far-field showed the potential for complex engineering of the nonlinear emission at the single meta-atom elements. Moreover, different SH patterns resembling a flower or a bird appeared in the far field of the structures, depending on the design parameters and pump polarization. Therefore, applying this concept to the single nano-structure, nonlinear holography was subsequently implemented in the case of metasurfaces [174].

**Figure 3:**
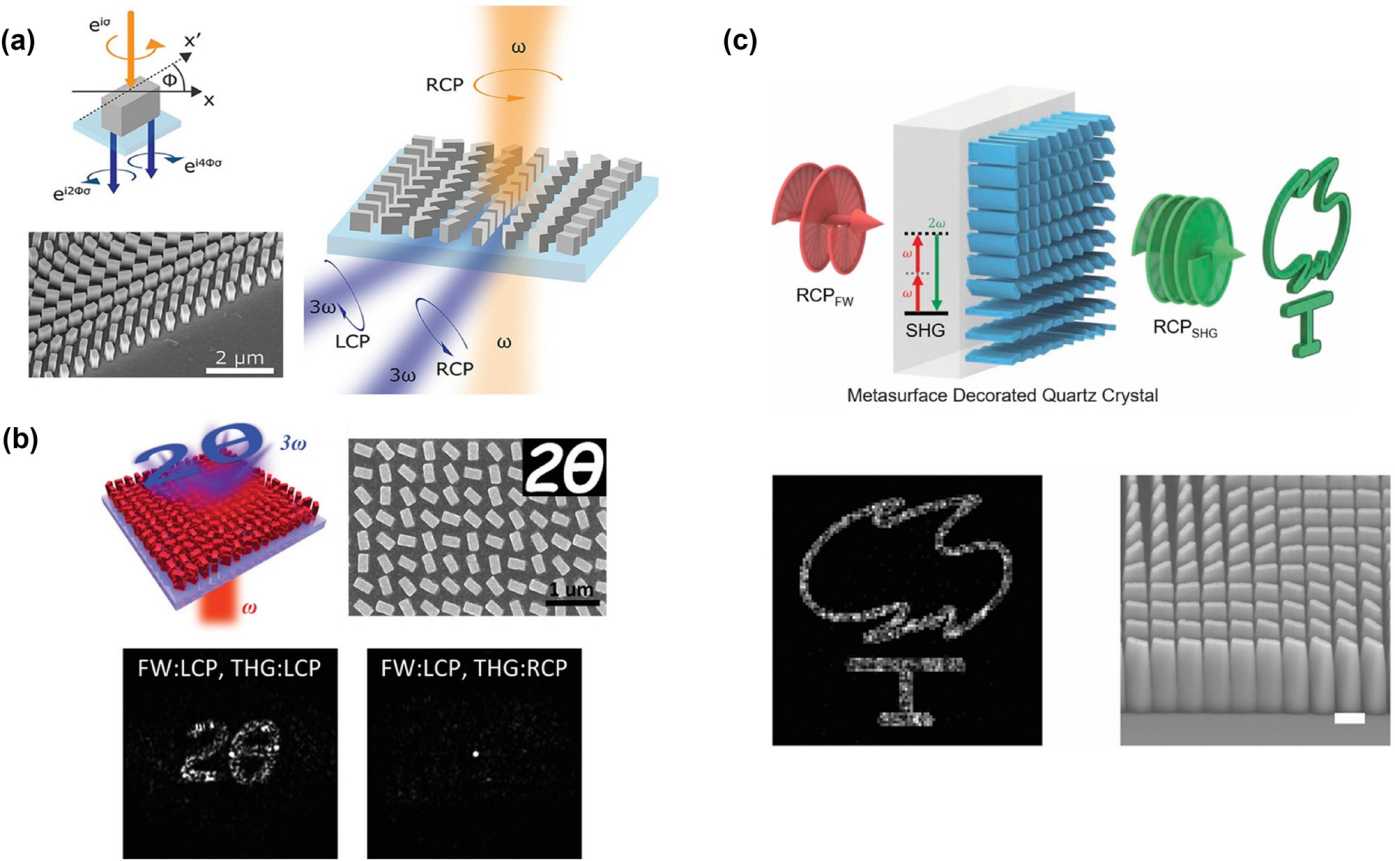
Nonlinear holography and image encoding with metasurfaces. (a) Schematic sketch and scanning electron microscopy (SEM) image of a geometric-phase silicon metasurface. The platform is excited with a right-circularly polarized (RCP) light and the nano-resonators that form the metasurface are displaced in order to encode the desired phase gradient at the RCP or left-circularly polarized (LCP) generated THG signal. Adapted from [64]. (b) Nonlinear holography based on nonlinear silicon nanofins metasurface. The top panels display the sketch of the proposed platform and a SEM image of the fabricated one. The bottom panels show the comparison between theoretical calculation and experimental results. The measured nonlinear hologram is obtained when the metasurface is shined with a fundamental wavelength (FW) signal with LCP light. For the co-polarized TH signal, a well-defined holographic pattern can be seen, whereas for cross-polarized TH signals, nothing can be seen. Adapted from [65]. (c) Illustration of the concept of generation of images with the silicon nitride metasurface on the quartz crystal for nonlinear wavefront engineering (top panel). Experimentally recorded SHG images from the device (bottom left panel). SEM image of the fabricated metasurface. Scale bar: 500 nm (bottom right panel). Adapted from [66].

In [64] a Pancharatnam–Berry (PB) phase approach to encode phase gradients and holographic images on silicon metasurfaces is reported. We note that the concept of PB phase metasurface (or geometric phase metasurface) allows for achieving a continuous phase variation by exploiting the rotational degree of freedom of the optimized meta-atom. In more details, by tuning the orientation angles of the silicon nanofins that form the proposed metasurface [64], it was possible to guarantee the desired phase profile of the emitted third-harmonic signal, see Figure 3(a). Hence, the results for the phase-gradient metasurface demonstrate that the spatial phase of the nonlinear signal can be controlled by the spatial orientation of identical meta-atoms. This concept can be used to encode more complex spatial phase profile into a metasurface as a function of the polarization state. In [64] the authors also demonstrated that metasurfaces, which have encoded the phase distribution of a computer-generated hologram, can provide two different nonlinear phases of the TH (third harmonic) light for two generated polarization states. Since both TH polarization states carry different phases and are orthogonal to each other, this design has been successfully employed for nonlinear multiplexed holograms. The dynamic switching between two TH images was achieved by switching the observed polarization from co-to cross-polarized with respect to the pump polarization.

More complex methods to expand the spatial and spectral holographic multiplexing have also been considered [171, 175–179]. For instance, in [175] it was proposed to exploit FWM nonlinear process in a metasurface composed of nano-hole arrays in a gold film for achieving amplitude computer-generated holograms, which carry different information at different frequencies. FWM was also shown in plasmonic [180, 181], hybrid [182], and all-dielectric metasurfaces [51]. Such nonlinear metasurfaces have great potential in high-security optical encryption, anti-fraud applications, and multicolor holography displays [168, 172]. Let us underline that the concept of nonlinear holograms not only inspires new imaging technologies but also provides a solid background for modulating the nonlinear optical waves. Indeed, as it has been studied in [65] the circularly polarized TH signal coming in the forward direction from the dielectric metasurface, composed of silicon nano-resonators with different in-plane rotational symmetries, carries the geometric phase, as predicted by the third harmonic generation selection rules. The nonlinear *k*-space holography by using the aforementioned metasurfaces has been successfully proved [65], see Figure 3(b).

Interestingly, in [183] the concept of asymmetric response has been introduced. In particular, the authors demonstrate that properly designed all-dielectric metasurfaces can generate different TH images in transmission for the two opposite illumination directions [183]. The working principle relies on interplay between magneto-electric coupling and nonlinear light–matter interaction. The resonators that form the metasurface consist of two layers of different materials with diverse refractive indices (silicon nitride and amorphous silicon) and they can generate light via THG processes with different intensity. Hence, the resonators have been optimized to generate different nonlinear signals to achieve completely different images for the two incident excitation directions [183]. In this context, the multiplexing capability may be reached by varying the direction of the incident light by properly designing metasurfaces whose nonlinear response is strongly asymmetric with respect to the pump direction. However, the possibility to have a platform with concurrently high nonlinear optical efficiency and powerful wave shaping is still difficult to realize. To overcome this limitation, in [66] it has been proposed to use a dielectric metasurface on top of quartz crystal to prove the efficient generation of nonlinear vortex beams and SH holographic images, as shown in Figure 3(c). This strategy may have important impacts in nonlinear light manipulation and metalens design. In [179] a multiplexed four-channel hologram based on THG in a chiral Au–ZnO hybrid metasurface, in which LCP- and RCP-components of TH-holographic images are formed independently and the TH signal from each pixel of the metasurface can be turned on or off by LCP or RCP excitation, has been experimentally demonstrated.

The authors of [184] proposed the PB-phase-based nonlinear plasmonic metasurface made of split-ring resonators with different orientations, which can realize 1 bit, 2 bit, and 3 bit coding at the THG, fundamental frequency (FF), and SHG; also, based on the same design nonlinear ultrathin flat metalens with different focal lengths for THG, FF, and SHG was demonstrated.

### 3.2 Nonlinear metalenses

Metalens is generally defined as an array of optical nanoresonators on a surface that is capable of manipulating the properties of the scattered light wavefront. Hence, metalenses are able to focus the electromagnetic radiation with a significantly more compact footprint with respect to conventional bulky lenses. By exploiting nonlinear responses in passive metasurfaces, the optical functionalities of such structures can be further enriched, leading to entirely new application areas. In this case, the functionality of the lens can be obtained by a proper spatially variant phase, which is simultaneously added to the nonlinear light emitted at each meta-atom on the surface. In particular, light from an object at the fundamental frequency is collected by the metalens, converted to the harmonic frequency, and projected to the image plane. The image plane is defined by the focal length, *f*, and the distance of the object to the lens. In a first approximation, the relationship of the required phase distribution Φ_*xy*_ (in the *xy* plane) and the distance *f* that metalens should guarantee to focus the light at can be expressed:

(2) $$\Phi_{xy}=2\pi /\lambda \left(\sqrt{\left(x^{2}+y^{2}+f^{2}\right)}-f\right),$$

where *λ* is the operating wavelength. The first implementations by using this approach were obtained with plasmonic meta-atoms. For example, in [60], one of the first demonstrations of SH metalens has been reported by using the concept of the nonlinear PB phase for SHG from plasmonic meta-atoms with threefold rotational symmetry. By adjusting the orientation angle of the meta-atom, the local phase of SHG can be continuously tuned over the entire phase range (0–2*π*), thereby resembling the desired phase profile of a lens for the SH frequency. Following this procedure, helicity-dependent nonlinear beam focusing and nonlinear imaging by using SHG at visible wavelengths have been successfully demonstrated along with keeping unaltered the propagation of the fundamental light [60].

**Figure 4:**
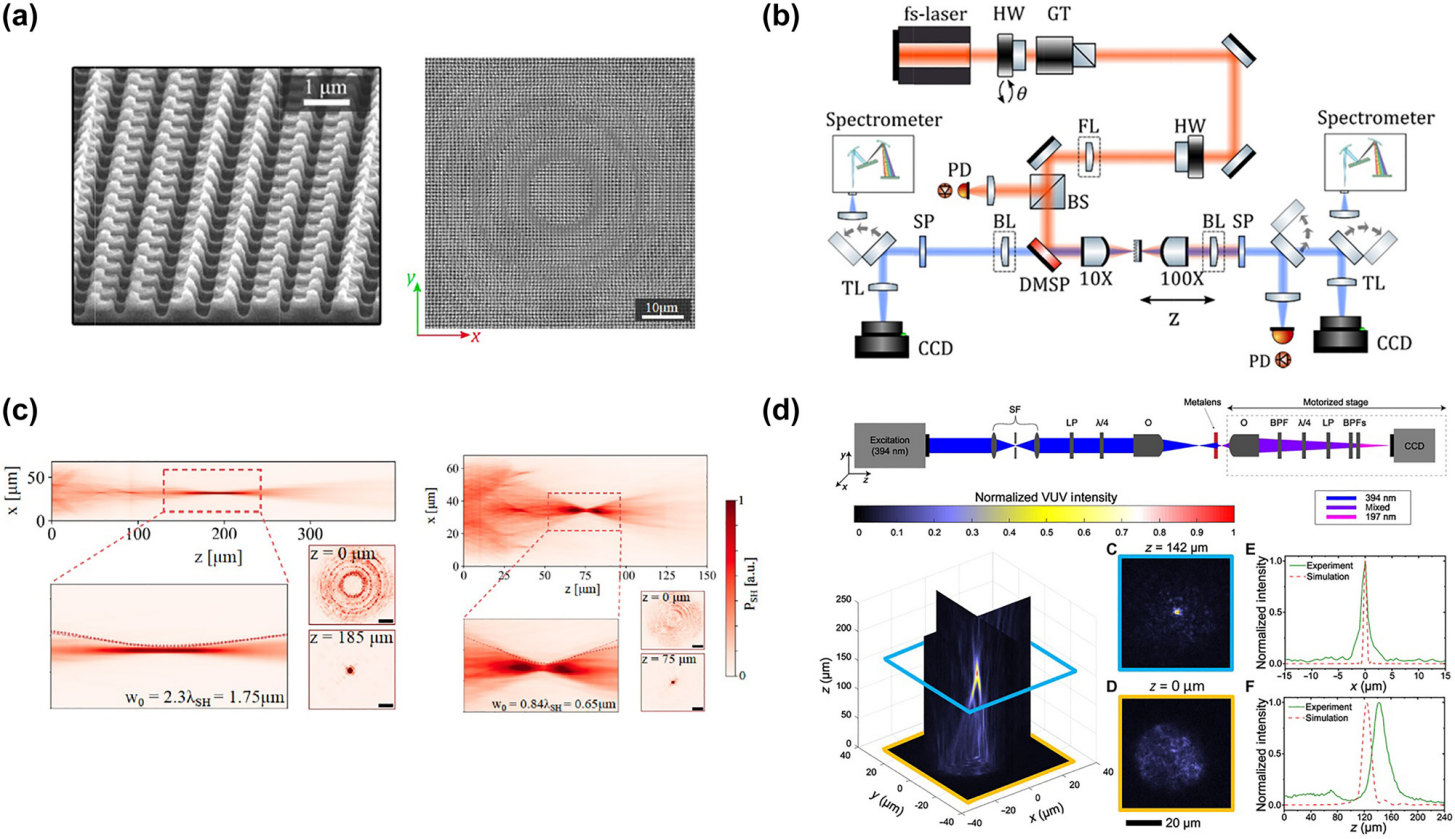
Nonlinear metalens with metasurfaces. (a) SEM views of nonlinear metalens made by AlGaAs nano-chairs. Adapted from [82]. (b) Experimental set-up for the characterization of nonlinear metalens. Adapted from [82]. (c) Experimental SHG focusing for two different metalens design parameters. Adapted from [82]. (d) The focusing experimental measurements of the VUV nonlinear metalens. Adapted from [56].

Recently, high refractive index dielectric nanoresonators have been proved to significantly increase the efficiency of nonlinear processes. Consequently, dielectric metalens implementations are soon followed. When encountering dielectric meta-atoms, the electromagnetic radiation can penetrate inside the nonlinear material and can therefore excite Mie resonances. In this framework, the geometrical aspect ratio of the nano-elements controls the wavefront shape via the nonlinear Huygens’ principle. When operated at a wavelength of 1550 nm, a TH metalens, composed of silicon nanopillars on a glass substrate, generated a TH emission in the visible range (i.e. 517 nm) [185]. The silicon pillars with elliptical basis were designed to concurrently guarantee almost the same level of TH magnitude and provide different phases in the range of 0–2*π* for different semiaxes values. The fabrication of metalenses of 200 × 200 μm^2^ footprint with *f* equal to 300 μm and 400 μm and TH generation conversion efficiency of the order of 10^−6^ is both theoretical and experimental proved [185]. However, the crucial milestone of realizing an efficient SH all-dielectric metalens has been possible by implementing a slightly more complex meta-atoms geometry also known as nano-chair [186], see Figures 4(a) and (b). In [82], making use of a look-up table relying on (100) AlGaAs nano-chair elements, the authors demonstrated SH beam steering and a Fresnel lenses metasurface, see Figure 4(c). In particular, a SHG efficiency of 1.3 × 10^−5^ is experimentally measured. This value is six orders of magnitude higher than the record SH one from plasmonic metasurfaces [39] and, importantly, one order of magnitude higher than the record value for TH in silicon metalens [185].

More recently, a metalens that generates (by nonlinear SHG) and focuses light in the vacuum ultraviolet (VUV) spectral region has been proved [56]. VUV light is useful for many applications from molecular spectroscopy to biomedical procedures and spans a range between 100 nm and 200 nm. However, the poverty of low-absorption VUV materials limits the realization of classical bulky VUV platforms. Various designs of VUV metasurfaces were introduced [56, 135, 187], [188], [189] to help overcome this drawback. In particular, in [56], a metalens made of zinc oxide nanoresonators has been demonstrated. This metalens converts the incident light (at a wavelength of 394 nm) into well-focused SH radiation (at 197 nm), eliminating the need for additional optical elements. The metalens has a diameter of 45 μm and it is composed of 8400 zinc oxide nanoresonators with triangular basis. The SH phase control with a range of 0–2*π* rad is obtained by rotating each meta-atom, providing the desired phase variation for VUV radiation. Figure 4(d) summarizes the focusing features of the latter platform. All the aforementioned results prove that, in a miniaturized optical system, metalenses can provide plenty of functions, including phase control and focusing. Further advanced optical performance can be reached by optimizing nano-resonators on the metalens surface. We strongly believe that metalenses will have significant impacts on different imaging systems, such as optical cameras, coherent tomography, and microscopy).

**Figure 5:**
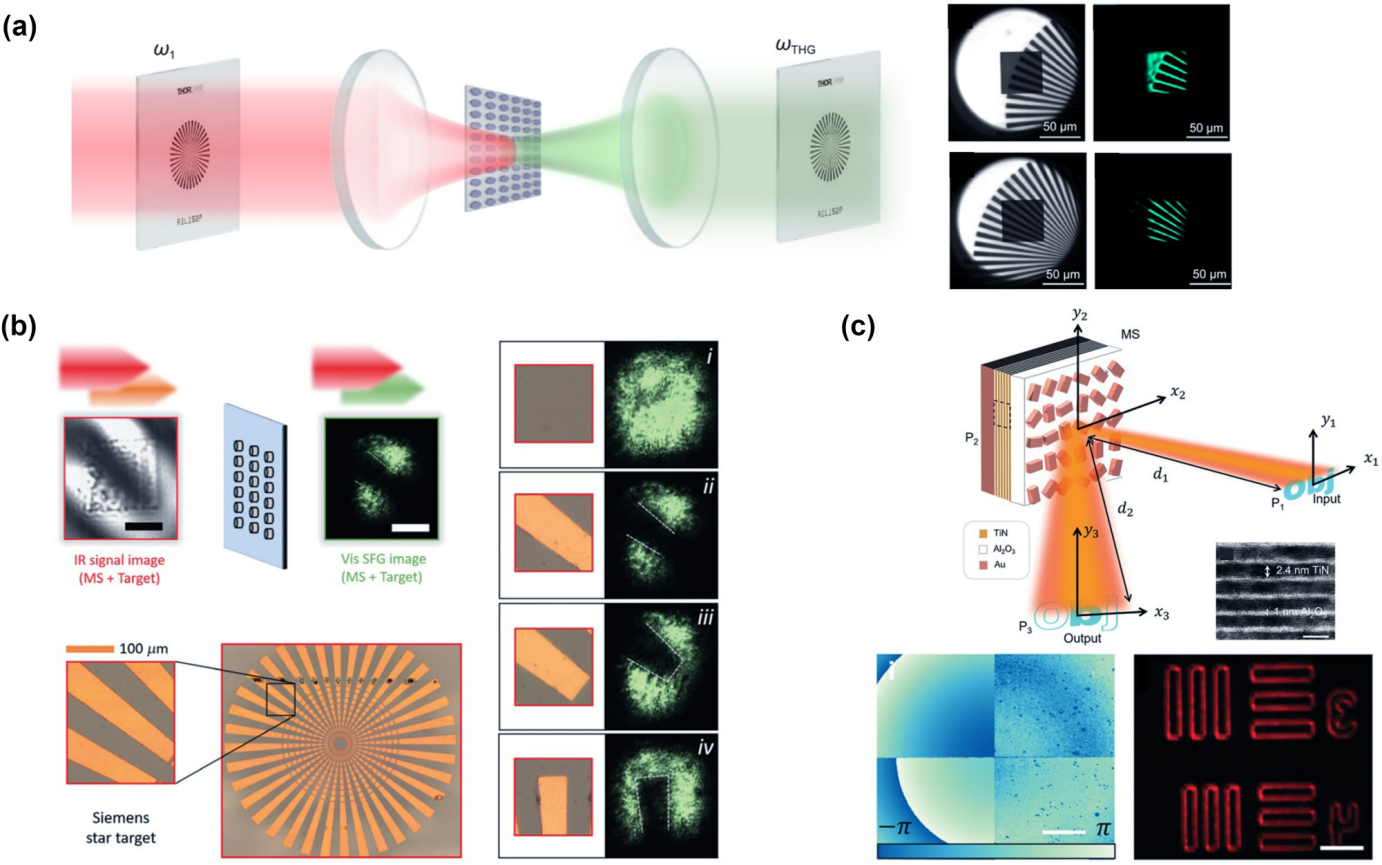
Upconversion imaging with nonlinear metasurfaces. (a) Left panel: conceptual sketch of TH generation and nonlinear image tuning through quasi-BIC MD resonators. The near-infrared signal passes through the target, then being converted into the visible signal via the metasurfaces, forming the target image on the CCD camera. The other four smaller panels display the experimental transformed visible images of the target via membrane metasurfaces under NIR light illumination. Adapted from [62]. (b) Infrared upconversion imaging using a GaAs metasurface through SFG processes. The IR signal beam passes through a target, which is imaged on the metasurface. By mixing the IR image with the pump beam is possible to obtain a visible image of the target (in the SFG beam), which is subsequently imaged by a lens onto a camera. The panels (i)–(iv) display the upconverted images for different transverse positions of the target. Adapted from [61]. (c) Top panel: schematic illustration of the nonlinear computational imaging metalens for edge-detection. Bottom panels: the phase image of the designed metalenses and the obtained edge image by using the designed metalens. Adapted from [63].

### 3.3 Nonlinear metasurfaces for image conversion and processing

Nonlinear metasurfaces represent a promising technology in the field of optics and photonics, with a strong potential for image conversion and processing. Indeed, by exploiting the nonlinear optical properties of their meta-atoms, nonlinear metasurfaces offer a wide range of possibilities for image manipulation, conversion, and processing beyond the limits of the traditional optics. One of the major applications of nonlinear metasurfaces in image processing is frequency conversion. In fact, these devices can efficiently translate input light from one frequency to another. For example, they can convert infrared images to visible images, enabling night vision applications or medical imaging [61, 62]. Moreover, the fast response of nonlinear metasurfaces, which is on the order of femtoseconds or picoseconds, makes them suitable for ultrafast imaging applications, including the study of dynamic processes in biology, materials science, and chemistry [38]. While nonlinear metasurfaces hold huge potential for image conversion and processing, their practical implementation and widespread adoption are still ongoing areas of research and development. Challenges include optimizing the efficiency of nonlinear processes, expanding the range of accessible wavelengths, and improving the scalability of manufacturing techniques. The extensive advancement and implementation of dielectric metasurfaces have been amply favored by the concept of bound-state-in-the-continuum (BIC). Although BIC is associated with the Q-factor going to infinity, the so-called quasi-BIC can be achieved by adding some asymmetry in the system. This quasi-BIC results in a reduced Q-factor, but allows for easier excitation of such modes with an external light source. The possibility of exciting a quasi-BIC in dielectric metasurface for efficient harmonic generation has suggested and further motivated the use of such structures for imaging applications.

For instance, the concept of quasi-BIC in silicon metasurfaces has been investigated for achieving dynamic spatial control of the nonlinear TH emitted signal [59]. The metasurface was composed of silicon nanodisks with a well-designed off-centered hole, which was necessary to break the symmetry and open a leaky channel for the BIC. The authors demonstrated the capability of such a platform to encode two different images into the metasurface by using resonances at different wavelengths or under distinct pump polarization. Similarly, in [62] the demonstration of the nonlinear conversion of an infrared image to the visible range has been proved in silicon dimer-hole membrane metasurfaces (Figure 5).

Metasurfaces made of As_2_S_3_ [190], Si [191], GaAs [192] or GaP [193] patterned [190], or grown [191–193] nanowires in transparent (PMMA [190]) and also flexible (PSMS [191–193]) polymers have been proposed as efficient tunable wavelength converters from IR to UV [190] and from IR to visible [191–193] via THG [190, 191] and SHG [192, 193] for imaging. We note that infrared upconversion imaging in dielectric metasurfaces has also been proved by using Gallium Arsenide (GaAs), in which lower-order nonlinear processes such as Sum Frequency Generation (SFG) can be efficiently realized. In [61] the authors demonstrated nonlinear wave-mixing of two infrared pumps (at 1530 nm and 860 nm), which generated a SFG upconverted emission in the visible range (around a wavelength of 550 nm). The incident excitation at 1530 nm was also named as the signal, while the beam at 860 nm was referred to be the pump. The metasurface, which was composed of GaAs cylindrical nanodisks embedded in a lower refractive index medium, was designed to be resonant at the three wavelengths of interest to maximize the nonlinear efficiency of the process. Moreover, the crystalline axis orientation [i.e. [110]] allows the generation of SFG signal in the vertical direction when both the pump and signal are normally incident to the metasurface. Notably, in [61] it is experimentally proved that when the signal beam carries the image of a target, the spatial information of the target is preserved in the nonlinear SFG process. Hence, the ultrafast nonlinear upconversion process allows infrared imaging with femtosecond temporal resolution. The reported achievements may open new perspectives for ultrafast imaging of chemical reactions in a conventional microscope device and for multi-color imaging at room temperature.

More recently, metasurfaces have also been tested for nonlinear computational imaging without requiring any additional optical components. One of the core operations in optical analog image processing is the edge-detection. In [63] a metalens made of nanoresonators with a static geometric phase and a nonlinear metallic quantum well layer is proposed as a good candidate for optical edge detection. Newly, in [194], by exploiting a simplified scenario of a uniform *χ* ^2^ thin sheet, the authors theoretically demonstrated the edge detection operation. The non-resonant nature of the nonlinear interaction permits edge detection over a broadband spectrum with ultra-high contrast and resilience to noise. All these results demonstrate that nonlinear flat optics can open concrete possibilities in numerous applications ranging from image processing to item recognition for computer vision.

## 4 The quantum light sources empowered by nonlinear metasurfaces

This section explores the advances in spontaneous pair photon generation from nonlinear metasurfaces. Recent progress has extended the quantum light source based on nonlinear metasurfaces from photon pair generation to spatially entangled photon pair generation, and even to the generation of complex quantum states based on pairwise coupling. In subsection 4.1, we first introduce the quantum–classical correspondence, connecting nonlinear metasurfaces to quantum light sources. We then discuss the recent works on improving the efficiency of spontaneous photon pair generation from nonlinear metasurfaces. In subsection 4.2, we demonstrate the efforts to produce entangled photon pairs via designing the resonant response of nonlinear metasurfaces. The entanglement of photon pairs is first exhibited in the momentum domain and then extended to the freedom of polarization. Finally, an approach called pairwise coupling can be applied to create the correlation between photon pairs in metasurfaces, which can prepare complex quantum states, such as cluster states.

### 4.1 Efficient spontaneous photon pair generation from nonlinear metasurfaces

Traditional production of photon pairs relies on the two-order and three-order nonlinear crystals utilizing spontaneous parametric down-conversion (SPDC) and spontaneous four-wave mixing (SFWM) processes. Aiming to confine the quantum light source into compact and integrated devices, fiber-based and waveguide-based optical systems have been well-investigated as the key integrated optical components over the last decades [8, 195–199]. The strong nonlinearities of fibers and waveguides play an important role in generating photon pairs via SPDC and SFWM. Under phase-matching condition, the conversion efficiencies of SHG based on the MoS_2_-functionalised fiber [200] and the heterogeneous titanium oxide/lithium niobate waveguide [15] can reach to 0.2 × 10^−3^ m^−2^ W^−1^ and 6.5 cm^−2^ W^−1^, which can enable the sufficient production of photon pairs. However, the phase matching condition and light propagation loss are significant factors in fibers and waveguides. They play a key role in determining the photon pair generation rate as part of the key performance for quantum light sources [199, 201]. Besides, the main loss in modern integrated waveguides comes from the scattering induced by the sidewall roughness inherently originated in fabrication processes [202]. Conversely, the phase matching condition is not superior in the sub-wavelength scale within nonlinear metasurfaces. The material absorption, as the main source of propagation loss in metasurfaces, can be minimized by selecting low absorption nonlinear materials in all-dielectric metasurfaces [203]. Additionally, nonlinear metasurfaces have unique abilities to empower their nonlinearities based on plasmonic resonances or multipolar Mie resonances via careful structural design. This approach does not rely on controlling propagating loss and the phase-matching term in fibers and waveguides. For these reasons, it is of great value to analyze how nonlinear metasurfaces can be applied as quantum light sources with their special functionalities.

The quantum–classical correspondence between SPDC and its reversed process (SFG) has been demonstrated, where the photon pair generation rate for SPDC can be predicted by utilizing the parameters of signal, idler incidents and generated far-field signal [206, 207]. This relation can be applied in quadratic nonlinear structures, including waveguides and metasurfaces, which can be expressed as below:

(3) $$\frac{1}{P_{p}}\frac{dN_{\mathrm{pair}}}{d\omega dt}=\frac{\omega_{i}\omega_{s}}{2\pi \omega_{p}^{2}}\eta_{n_{s}n_{i}}^{\mathrm{SFG}}\left(\omega_{s},\omega_{i}\right)$$

**Figure 6:**
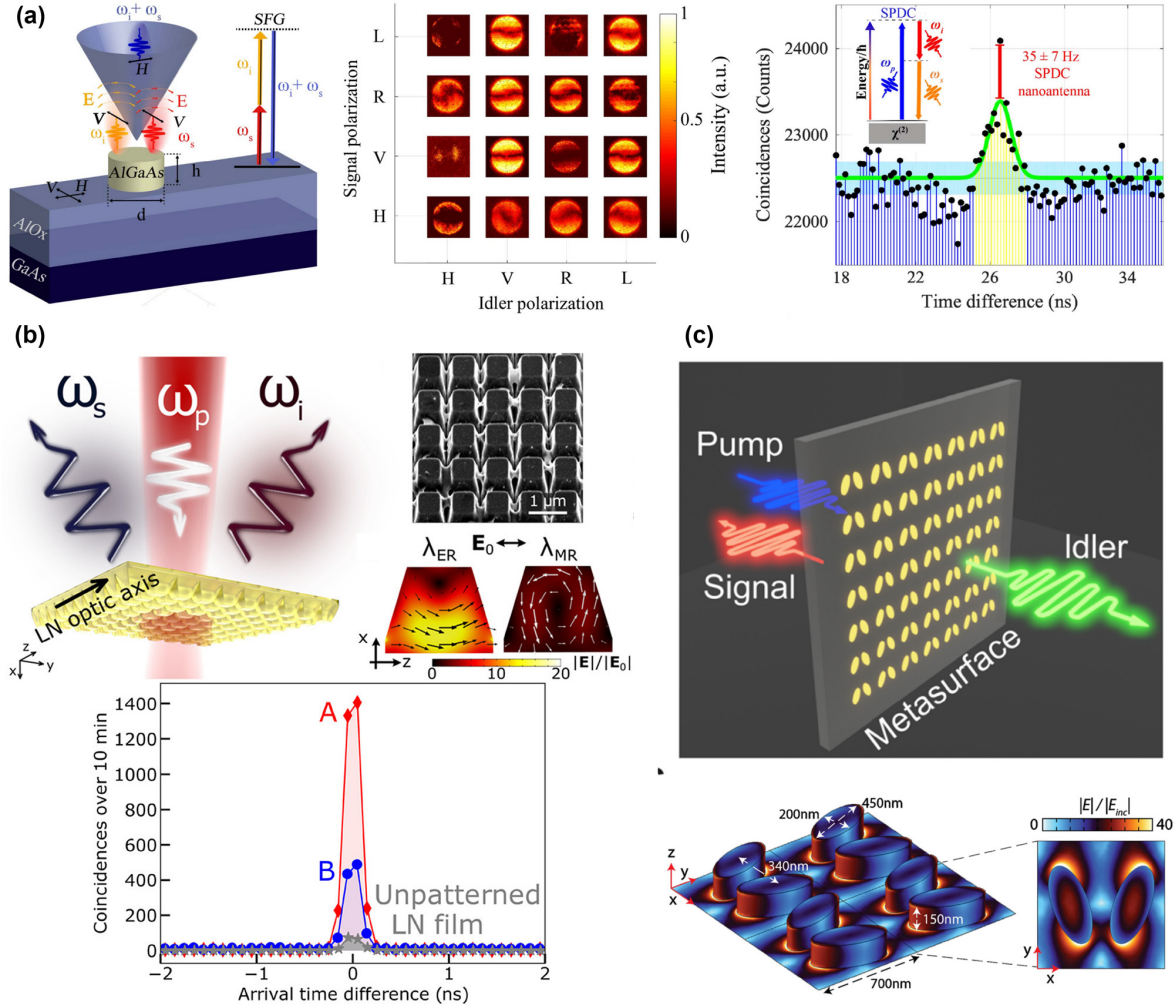
Spontaneous photon pair generations enhanced via resonant metasurfaces. (a) Left: The schematic of the SFG process based on an AlGaAs nanoantenna. Middle: Measured reflected SFG emission patterns in *k*-space for different polarization combinations. Right: Coincidence histograms of degenerate SPDC from an AlGaAs nanoantenna. Adapted from [73]. (b) Top left: The schematic of SPDC from a lithium niobate metasurfaces. Top right: Scanning electron microscopy (SEM) image of a fabricated metasurfaces and electric field distributions inside a nanoresonator at the electric resonances (*λ*_ER_) and magnetic resonances (*λ*_MR_). Bottom: Coincidence histograms of degenerate SPDC from two different metasurfaces, shown by red diamonds and blue circles, respectively. Adapted from [204]. (c) Top: SPDC in a metasurfaces with the signal and idler photons emitted in opposite directions. Bottom: The shape and dimensions of the unit cell and the electric field distribution. Adapted from [205].

Here, *P*_*p*_ is the pump beam power for SPDC, d*N*_*pair*_/d*ω*d*t* is the photon pair generation rate per unit signal frequency, and $\eta_{n_{s}n_{i}}^{\mathrm{SFG}}\left(\omega_{s},\omega_{i}\right)=P_{\mathrm{SFG}}/P_{s}P_{i}$ is the conversion efficiency of SFG. *ω*_*s*_, *ω*_*i*_, and *ω*_*p*_ are the frequencies of signal, idler, and pump beams, respectively. Such a correlation between classic and quantum allows for estimating the photon pair generation rate of SPCD via the conversion efficiency of classical SFG. This correlation also makes the connection from classical nonlinear metasurfaces to quantum metasurfaces. All the approaches that have been applied to enhancing SHG and SFG in nonlinear metasurfaces are also beneficial for designing metasurfaces-based quantum light sources. In [73], the degenerated photon pair generation from an AlGaAs nanoantenna has been experimentally demonstrated (shown in Figure 6(a)). Via designing the Mie resonances at the pump and photon-pair wavelengths, the photon pair generation rate reaches up to 35 Hz, which is 1 order of magnitude higher than conventional on-chip or bulky photon-pair sources after normalization. There is also an attempt to theoretically infer the polarization state of correlated photon pairs using the quantum–classical correspondence. It illustrates the potential in nanostructures that one can deduce the quantum state of the polarization-entangled photon pair by calculating the conversion efficiencies of the reversed SFG processes under the incidents with different signal and idler polarizations. Moreover, the application is extended from the single nanoantenna to metasurfaces. The lithium niobate metasurfaces supporting electric and magnetic Mie resonances have been experimentally revealed as a quantum light source via SPDC with degenerated photon-pair generation rate of 5.4 Hz, which is 20 times higher than the unpatterned lithium niobate film shown in Figure 6(b) [204]. Apart from utilizing the semiconductor materials, the nonlinear plasmonic metasurfaces are felicitous to be exploited in photon pair generation [208]. It has been theoretically demonstrated that plasmonic metasurfaces based on silver nanostripes combined with a lithium niobate film can be an efficient non-degenerate SPDC source. Additionally, the BIC-enhanced nonlinear metasurfaces can significantly improve the generation rate of the photon pair for SPDC. In [209], the hyperbolic topology of the transverse phase matching is constructed by two quasi-BICs, enabling the orders-of-magnitude increase of the nondegenerate photon pair rate and spectral brightness, theoretically. The experimental contribution is achieved using GaP metasurfaces with remarkable emission directivities at the vicinity of quasi-BIC resonance (Figure 6(c)) [205]. The photon pair generation rate is enhanced 67 times compared to an unpatterned film of the same thickness and material. More importantly, the bidirectional emission is observed: photons are mostly emitted backward at the vicinity of quasi-BIC resonance, while their partners are partially emitted forwards. This observation indicates the capabilities of nonlinear metasurfaces, which can not only generate photon pairs, but also split and filter the bi-photons. It shows the possibility of designing multifunctional metasurfaces, for example, as a quantum light source and also a quantum operator manipulating the quantum state of produced photon pairs. In this subsection, we report the recent progress of nonlinear metasurfaces as quantum light sources with enhanced photon pair generation rates, from single nanoantennas to metasurfaces, from all-dielectric to plasmonic metasurfaces, from ED and MD Mie resonances to quasi-BICs empowering remarkable light–matter interaction. In the end, we discuss the potential of the multifunctional metasurfaces combining the capabilities of generating photon pairs and manipulating their quantum state into one integrated nanodevice.

### 4.2 Entangled photon pair generation from nonlinear metasurfaces

**Figure 7:**
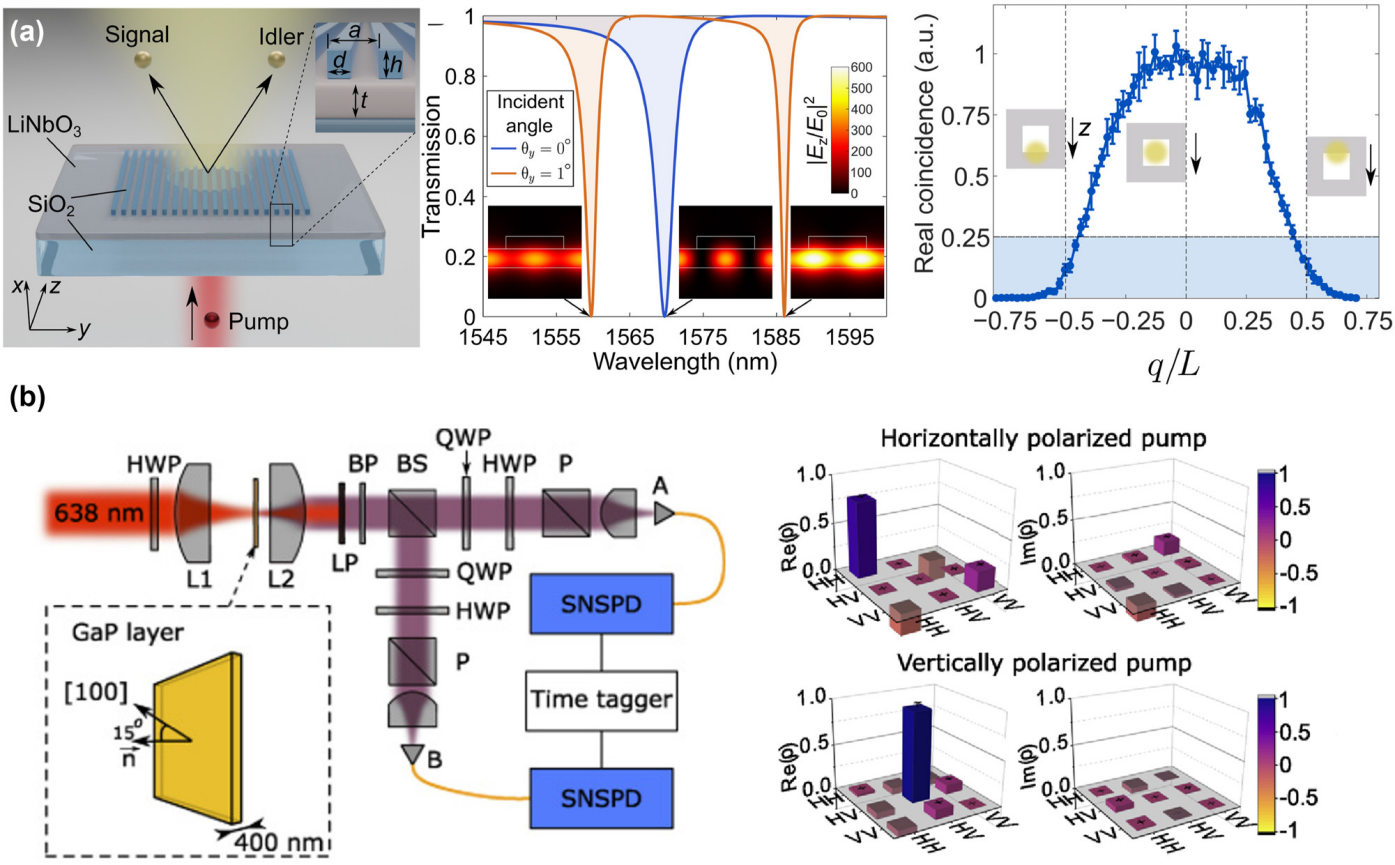
Entangled photon pair generations enhanced via resonant metasurfaces. (a) Left: The schematic of spatially entangled signal and idler photon generation from a lithium niobate thin film covered by an SiO_2_ grating and pumped by a continuous laser. Middle: Simulated transmission spectra of the metasurfaces, showing a single guided mode resonance at normal incidence and two resonances with a nonzero incident angle. Right: The real coincidence rate of collected photon pairs versus the aperture position, normalized to the maximum value. Adapted from [74]. (b) Left: The schematic of experimental setup for measuring the quantum state of entangled photon pairs encoded by polarization states. The sample is fabricated with its normal at 15° to the [100] crystalline direction of GaP crystal. Continuous-wave pump focused by lens L1 into the GaP film; photon pairs are collected by lens L2 and filtered from the pump by long-pass filters LP and bandpass filter BP. Nonpolarizing beam splitter BS sends the photons into arms A and B, each containing a quarter-wave plate (QWP), a half-wave plate (HWP), a polarizer (P), and a superconducting nanowire single-photon detector (SNSPD). A time tagger builds a histogram of arrival time differences. Right: Real and imaginary parts of the density matrix *ρ* of the photon pairs generated by the H- (V-) polarized pump, indicating that photon pairs are shifted from the highly entangled state with the horizontally polarized pump to nearly disentangled with the vertically polarized pump. Adapted from [79].

In the modern quantum technical treatments of spontaneous photon generation, the generation rate is not the only index to be concerned with. The entanglement of produced photon pairs is also essential in the quantum industry. To date, how to construct highly entangled qubits such as bell states and cluster states has been notably demanded [210–215]. A vast range of platforms including nonlinear crystals, fibers, waveguides, microcavities, and quantum dots have been widely used to generate photonic entanglement in all degrees of freedom (frequency, time, polarization, spatial mode, and orbital angular momentum) [216, 217], including polarization-entangled [218], momentum-entangled [219], time-bin-entangled [220], and energy-time-entangled [221] photon pairs. Nowadays, nonlinear metasurfaces, with the ability to engineer the wavefront and the potential on integrating the manipulation of quantum states with producing a massive amount of correlating photon pairs, aim to construct the desired entangled quantum states. It has been demonstrated that metasurfaces incorporating a nonlinear thin film of lithium niobate can generate spatially entangled photon pairs through SPDC, as revealed in Figure 7(a) [74]. Via adding the grating on top of the film, the continuous translational symmetry is broken to excite the guided-mode resonances inside the lithium niobate layer. The guided mode with even field distribution forms a BIC under the protection of rotational symmetry. When breaking the rotational symmetry by changing the incident angle, two high-Q resonances appear at both sides of the resonance at Γ point, forming a topological frequency splitting. This kind of splitting supports the strong entanglement of photons in space, for satisfying the transverse phase matching of SPDC while the longitudinal matching requirements are significantly eased because of an ultra-thin thickness. As a result, the spatial correlations of photon pairs and the violation of classical Cauchy–Schwarz inequality (CSI), as a criterion of spatial antibunching and multimode entanglement, are experimentally detected. Moreover, the photon pair generation rate is around 450 times stronger than unpatterned structures, exhibiting a purely linear polarization state of the photons with a high extinction ratio of above 99 percent – a nearly pure polarization state. This work indicates that via designing the resonance with transverse wave numbers satisfying the transverse phase matching, nonlinear metasurfaces can be the source of highly entangled photon pairs in space.

To date, the entanglement of photon pairs from nonlinear metasurfaces via SPDC has been experimentally achieved in the momentum domain. The freedom of the polarization state is the next set required to be complemented. The polarization entanglement of the photon pairs can be tuned by changing the pump polarization in “flat-optics” [79]. demonstrates the generation of degenerate photo pairs via SPDC from 400-nm GaP film with its normal at 15° to the [100] crystalline direction, where the polarization state of photon pairs can be tuned via changing the pump polarization (shown in Figure 7(b)). Through independently selecting different polarization states in each beam splitter output port, the polarization state and the density matrices *ρ* of the generated pairs can be characterized by utilizing the two-qubit polarization tomography. From the reconstructed density matrices, it has been clearly demonstrated that the photon pairs are highly entangled with the horizontally polarized pump and nearly disentangled with the vertically polarized pump. By changing the pump polarization, the photon pairs are shifted from highly entangled states to almost disentangled states. These results indicate the capability of nonlinear metasurfaces on constructing numerous entangled photon pairs with manipulated polarization states, which can be encoded as qubits in quantum computing. However, the direct experimental achievement of constructing Bell states, as the maximally entangled two-qubit state, via nonlinear metasurfaces is remaining unimplemented.

Apart from the entanglement between one photon pair, the coupling between different photon pairs has been recognized as an essential phenomenon to create complex quantum states to obtain multiple qubits in quantum computing, such as cluster states. The SPDC from resonant nonlinear metasurfaces based on high-Q quasi-BIC resonances is revealed, including a feasible approach that can generate multifrequency quantum states, including cluster states [75]. A nondegenerate photon pair is path-correlated between each other after the beam splitter. These nondegenerate photon pairs can be connected to create multifrequency pairwise correlated qubits by emitting one photon from each pair at the resonant wavelength |*λ*_res_⟩. To do so, it requires the photons at the resonant wavelength from each pair to be indistinguishable. Nonlinear metasurfaces can be designed to be divided into several pixels. Each pixel can generate nondegenerate photon pairs with mutually coherent pumps, which allows the realization of pairwise correlated qubits. When each photon pair is bell states (maximally entangled states), this strategy called pairwise coupling can be utilized in generating cluster states. This increases the number of qubits that nonlinear resonant metasurfaces can construct via SPDC, and expands its application range in the quantum computing field.

## 5 Outlook and conclusion

The field of nonlinear metasurfaces has been widely explored in the past decade. We present an overview of the recent progress of nonlinear metasurfaces for imaging, quantum, and sensing applications. Despite significant progress has been made in these domains, there is still a rich variety of opportunities awaiting further exploration:

### 5.1 Outlook on quantum applications of nonlinear metasurfaces

The utilization of photonic qubits in optical platforms offers promising opportunities for realizing large-scale quantum processors and long-distance quantum communications, leveraging photons’ room-temperature stability and long coherence time [222, 223]. Nonlinear metasurfaces, with their ability to precisely modulate the polarization, phase, and amplitude of photons, hold significant potential in preparing, manipulating, and measuring quantum states with high fidelity.

In previous sections, we have discussed the creation of a wide variety of photonic quantum states, including single and entangled photons by harnessing the nonlinear optical effects such as SPDC and SFWM in nonlinear metasurfaces (see Section 4). However, the momentum conservation limitation for participating photons restricts the versatility and efficiency of the resulting quantum states. To overcome this limitation, resonant nonlinear metasurfaces have been proposed to enable the creation of versatile and complex quantum states [75]. For instance, the use of cross-Kerr nonlinear metasurfaces has been proposed for high-quality Bell state preparation [224]. These metasurfaces naturally generate various polarization-entangled Bell states over a broad range of wavelengths and emission directions, requiring little to no engineering effort [224]. This advancement holds great potential for the development of quantum communication techniques, particularly quantum key distribution, where Bell states serve as the initial shared key [225]. Additionally, the generation of photon–plasmon quantum states using nonlinear hyperbolic metamaterials [206] indicates the potential application of nonlinear metasurfaces in cross-platform quantum technologies, which involve quantum states composed of multiple physical entities and enable the creation of unique spatial patterns or optical modes that carry quantum information.

In terms of quantum operations, the interaction between photons poses challenges due to their bosonic nature. However, nonlinear metasurfaces offer the potential to enhance photon interactions, making them promising candidates for engineered quantum gates. One advantage of using metasurfaces as quantum gates is their tunability through external optical stimuli, enabling dynamic manipulation of photonic quantum states for quantum information processing purposes [226, 227]. This tunability eliminates the need for reconfiguration and complicated experimental setups. For instance, tunable quantum metasurfaces with phase-changing materials enable polarization tuning at optical frequencies [228]. By influencing the properties of the nonlinear material with one photon and subsequently interacting with other photons, entangled gate photons can be achieved [229]. Furthermore, quantum nonlinear metasurfaces, such as subwavelength arrays of ultracold atoms, form a quantum metasurface that enables strong photon–photon interactions. This interaction can convert an incoming classical beam into strongly correlated photonic states with minimal photon losses, providing opportunities to explore quantum many-body phenomena in two-dimensional systems of strongly interacting photons [230]. The compactness and scalability of these metasurfaces are crucial for building practical quantum computing systems with a large number of qubits.

Moreover, the thin and planar design of metasurfaces allows for easy integration with other elements of a quantum system, such as waveguides, detectors, or quantum memories, to create more complex quantum circuits and enhance the overall flexibility of the system [231, 232]. In solid-state-based quantum computing platforms, such as quantum dots or nitrogen-vacancy centers, nonlinear metasurfaces can interact with embedded quantum emitters, enabling entanglement between the emitters and photons and facilitating the implementation of gate operations that couple different components of the quantum system [233]. Metasurfaces integrated with photonics can facilitate on-chip trapped ion and atoms quantum computing by enabling efficient optical interfacing and enhancing the performance of on-chip optical components [234]. With the help of metasurfaces, strong light–matter interactions at the quantum level can be achieved [235]. This opens up possibilities for implementing quantum gates and quantum memories, which are critical components in quantum information processing.

Nonlinear metasurfaces also find wide applications in high-dimensional quantum information encoding and processing [236], where the multiple spatial or polarization modes of photons can enhance encoding capacity and processing capability of photonic qubits. Nonlinear metasurfaces can also enable holography with multidimensional optical data storage [172].

In terms of measurements, linear metasurfaces can be designed to project photons to different directions based on their polarizations. This approach inherently provides uniformly sampled measurements over the commonly used Pauli measurement bases [78]. Unlike superconducting or trapped ion quantum processors where changing the measurement basis requires introducing additional gates, metasurface-based detectors guarantee that the state information can be extracted from the photon counts without reconfiguring the optical apparatus. Furthermore, this measurement approach is scalable since the complexity of metasurface design scales linearly with the number of qubits [237]. With fabrication precision improved, the polarization projection basis of metasurfaces can be designed to follow a quantum *t*-design, which has been proven to be the optimal measurement scheme for photonic qubits [238]. When combined with error mitigation techniques, metasurfaces can be used as devices implementing robust randomized measurements, which enables efficient estimation of properties of the photonic qubits.

The response of metasurfaces to incoming photons may produce a measurable signal that signifies the presence of the photon. When combined with superconducting nanowire single-photon detectors instead of traditional avalanche photodiodes (APDs), the detection efficiency can be improved, and the time-domain resolution is sufficient to resolve photon counts [239]. These factors make metasurfaces ideal candidates for versatile photonic qubit detectors. The sensitive response feature of metasurfaces enhances the sensitivity of quantum sensors, enabling the detection of weak signals with higher precision. This has applications in quantum metrology, where precise measurements are necessary, such as in gravitational wave detection or magnetic field sensing [240]. By engineering a metasurface to interact with specific wavelengths of light or photons relative to the behaviors of biological systems, there exists the potential to advance the development of innovative bioimaging and quantum biosensing tools [241, 242].

In summary, nonlinear metasurfaces hold significant promise in the quantum field. Their ability to prepare and manipulate quantum states, serve as engineered quantum gates, and enhance quantum measurement and detection makes them versatile and valuable tools.

### 5.2 Nonlinear sensing with metasurfaces

Optical sensors exploit different light–matter interactions in biological samples, detecting optical parameters like absorption, emission, scattering, dielectric constant, and chirality, etc. These interactions include both linear (*e.g.* absorption, scattering, interference) and nonlinear (*e.g.* Raman scattering, fluorescence) processes. Linear interactions give rise to a wide range of biosensing techniques including absorption spectroscopy, interferometry, refractometry, circular dichroism (CD) spectroscopy, dynamic light scattering (DLS), etc. Nonlinear interactions, including Raman scattering, fluorescent emission and up/down conversions, lead to techniques such as Raman spectroscopy, fluorescence microscopy, and SHG microscopy. As discussed in previous sections, metasurfaces possess unique ability to manipulate the amplitude, phase and polarization of the light at subwavelength scales. Through specific design, metasurfaces can selectively enhance light–matter interactions in the biosensing, enabling high-sensitivity sensing at low concentrations. This unique feature positions metasurfaces as ideal candidates for advanced biosensors. Over the past decades, metasurfaces have revolutionized the optical biosensing field, promoting both linear and nonlinear biosensing [68, 81, 243].

In the linear realm, metasurfaces have significantly improved refractive-based biosensing. Their ability of being highly sensitive to the environmental refractive index allows them to detect biomolecules at ultra-low concentrations, pushing the limits of the detection several orders of magnitude compared to the traditional methods. Metasurfaces have also proven to be highly effective in improving the sensitivity of chiral biomolecular sensing [244–247], which is important for detecting supra-structural chirality of proteins or DNA. Besides amplifying the optical signals, metasurfaces have overcome the bottleneck of optical devices, for example, optical tweezers. Traditional optical tweezers face restrictions in manipulating objects smaller than hundreds of nanometres due to the diffraction limit. Metasurfaces provide highly confined optical fields smaller than the diffraction, making it possible to trap nanoscale objects. In particular, the concept of self-induced back-action (SIBA) trapping has emerged, enabling the trapping of single proteins (several nanometres in size) for a long duration [248, 249]. Analyzing the scattering signals from the nanostructure provides valuable information on protein folding states [250]. This aperture-based nanostructure makes it possible to size proteins, detect protein conformational changes, and follow the protein-protein interactions [251–254].

Nonlinear optical processes offer effective separation in the signal frequency from the excitation light, enabling low-background detection and a high signal-to-noise ratio in biosensing. Metasurfaces play a crucial role in enhancing the nonlinear biosensing processes, making significant contributions to the technologies such as surface-enhanced Raman scattering (SERS), and single-molecule fluorescent biosensing.

Raman spectroscopy detects the inelastic light scattering raised from molecular vibrations, providing a unique “fingerprint” of the molecules based on the energy shift of the scattered photons corresponding to the chemical bonds [255]. However, due to its inherently weak activity – one in 10^8^ of the incident photons undergoes spontaneous Raman scattering [256] – Raman spectroscopy often requires either long scanning time or highly concentrated sample. The invention of surface-enhanced Raman spectroscopy (SERS) [257], which greatly improved the Raman efficiency, makes it widely applicable in biosensing. Since the Raman signal enhancement is proportional to the fourth power of the local electric field, plasmonic metasurfaces could provide extremely strong near-field enhancement due to the localized surface plasmonic resonance [258–260]. Plasmonic metasurfaces have been demonstrated to enhance Raman scattering of the order of 2 × 10^5^, enabling quantitative SERS at the single molecular level (SM-SERS) [261], or even single amino acid discrimination [262].

Fluorescent-based biosensing is another approach that enables the detection of low-concentration species. This technique relies on the fluorescence emission from the biological sample or the fluorophores that are attached to the biosamples of interest. Fluorescence biosensing, due to its highest sensitivity, led to the development of various single-molecule biosensing techniques such as fluorescence correlation spectroscopy (FCS), fluorescence resonance energy transfer (FRET), and super-resolution microscopy. Both metallic and dielectric metasurfaces demonstrate their great contribution to the fluorescent labelled sensing, either amplifying the excitation electric field or enhancing the emission efficiency. Metasurfaces Metallic metasurfaces have proved to enhance the fluorescent intensity up to three orders of magnitude [263–268]. However, plasmonic materials have the high Ohmic loss, limiting their practical use. Dielectric metasurfaces, on the other hand, offer low loss and minimal heat generation, making them highly suitable for enhancing the fluorescence signal of the bio sample [269–274]. Metasurfaces have significantly improved the sensitivity of fluorescent sensing, achieving the level as low as pg/mL (fM) [275–278] and further pushed limit of detection (LoD) to 5.86 aM [279]. Another remarkable application of metasurfaces in fluorescence microscopy is generating metalenses that create specific beam shapes and break optical limitations for high-resolution microscopy [280, 281].

SHG biosensing exploits the intrinsic nonlinear properties of biomaterials, making it highly suitable for characterizing specific molecule interactions at the surface [282]. SHG microscopy techniques have been instrumental in examining biological interfaces and interactions with exceptional spatial resolution. By using the plasmonic silver nanohole structure, scientists have improved the resolution of SHG signatures down to single molecule detection and visualized 3D orientation of individual rhodamine 6G molecule [283].

Until now, the majority of nonlinear biosensing using metasurfaces, as mentioned above, has relied on the intrinsic nonlinear properties of specific biological samples. However, this dependence on material’s intrinsic properties limits its application to certain materials and may require specialized expertise, such as site-specific labelling. To overcome these limitations, one potential direction is to design nonlinear metasurfaces capable of converting linear light–matter interaction signals into nonlinear signals. For instance, nonlinear metasurfaces can be designed so that the nonlinear efficiency is sensitive to the frequencies. By utilizing the refractive index sensing principle, the presence of biomolecules can induce a shift in resonance, thereby altering the metasurface’s nonlinear emission. By detecting the nonlinear signals of the metasurface, such as SHG, SFG or FWM, one can effectively detect the linear light–matter interaction processes in the biological sample, similar to the concept of nonlinear imaging with metasurfaces [260]. This approach has the potential to broaden the accessibility of metasurface-assisted nonlinear biosensing or bioimaging for a wider range of biological samples.

In conclusion, the field of nonlinear metasurfaces is rapidly advancing and evolving into a prominent frontier within photonics research. This evolution is marked by a shift from fundamental research to the active development of practical applications. The thriving research in nanophotonics and nanotechnology serves as a fertile ground for the continued integration of nonlinear metasurfaces with other research areas. Exploring complementary directions such as reconfigurable and programmable metasurfaces, as well as investigating the intersection of nonlinear metasurfaces with promising fields like topological photonics to locally engineer the field properties, presents a promising opportunity for extending the capabilities of current nonlinear metasurfaces in imaging, quantum, and sensing applications through the development of dynamic and multi-functional active nonlinear meta-devices.

## References

[1] Franken P. A., Hill A. E., Peters C. W., Weinreich G. (1961). Generation of optical harmonics. *Phys. Rev. Lett.*.

[2] Kravtsov V., Ulbricht R., Atkin J. M., Raschke M. B. (2016). Plasmonic nanofocused four-wave mixing for femtosecond near-field imaging. *Nat. Nanotechnol.*.

[3] Deka G., Sun C.-K., Fujita K., Chu S.-W. (2017). Nonlinear plasmonic imaging techniques and their biological applications. *Nanophotonics*.

[4] Frischwasser K., Cohen K., Kher-Alden J., Dolev S., Tsesses S., Bartal G. (2021). Real-time sub-wavelength imaging of surface waves with nonlinear near-field optical microscopy. *Nat. Photonics*.

[5] Reid M., Drummond P. (1988). Quantum correlations of phase in nondegenerate parametric oscillation. *Phys. Rev. Lett.*.

[6] Kumar P. (1990). Quantum frequency conversion. *Opt. Lett.*.

[7] Langrock C., Diamanti E., Roussev R. V., Yamamoto Y., Fejer M. M., Takesue H. (2005). Highly efficient single-photon detection at communication wavelengths by use of upconversion in reverse-proton-exchanged periodically poled linbo 3 waveguides. *Opt. Lett.*.

[8] Zhao J., Ma C., Rüsing M., Mookherjea S. (2020). High quality entangled photon pair generation in periodically poled thin-film lithium niobate waveguides. *Phys. Rev. Lett.*.

[9] Mesch M., Metzger B., Hentschel M., Giessen H. (2016). Nonlinear plasmonic sensing. *Nano Lett.*.

[10] Verma M. S., Chandra M. (2020). Nonlinear plasmonic sensing for label-free and selective detection of mercury at picomolar level. *ACS Sens.*.

[11] Yu R., Cox J. D., De Abajo F. J. G. (2016). Nonlinear plasmonic sensing with nanographene. *Phys. Rev. Lett.*.

[12] Agrawal G. P. (2011). Nonlinear fiber optics: its history and recent progress. *JOSA B*.

[13] Agrawal G. P. (2000). Nonlinear fiber optics. *Nonlinear Science at the Dawn of the 21st Century*.

[14] Lin Q., Painter O. J., Agrawal G. P. (2007). Nonlinear optical phenomena in silicon waveguides: modeling and applications. *Opt. Express*.

[15] Luo R., He Y., Liang H., Li M., Lin Q. (2019). Semi-nonlinear nanophotonic waveguides for highly efficient second-harmonic generation. *Laser Photonics Rev.*.

[16] Boyd R. W. (2008). *Nonlinear Optics*.

[17] Espinola R. L., Dadap J. I., Osgood R. M., McNab S. J., Vlasov Y. A. (2004). Raman amplification in ultrasmall silicon-on-insulator wire waveguides. *Opt. Express*.

[18] Chowdhury A., Ng H. M., Bhardwaj M., Weimann N. G. (2003). Second-harmonic generation in periodically poled gan. *Appl. Phys. Lett.*.

[19] Mizuuchi K., Yamamoto K., Kato M., Sato H. (1994). Broadening of the phase-matching bandwidth in quasi-phase-matched second-harmonic generation. *IEEE J. Quantum Electron.*.

[20] Hum D. S., Fejer M. M. (2007). Quasi-phasematching. *C. R. Phys.*.

[21] Neshev D. N., Miroshnichenko A. E. (2023). Enabling smart vision with metasurfaces. *Nat. Photonics*.

[22] Chen H.-T., Taylor A. J., Yu N. (2016). A review of metasurfaces: physics and applications. *Rep. Prog. Phys.*.

[23] Genevet P., Capasso F., Aieta F., Khorasaninejad M., Devlin R. (2017). Recent advances in planar optics: from plasmonic to dielectric metasurfaces. *Optica*.

[24] Estakhri N. M., Alù A. (2016). Recent progress in gradient metasurfaces. *JOSA B*.

[25] Zhao Y., Liu X.-X., Alù A. (2014). Recent advances on optical metasurfaces. *J. Opt.*.

[26] Li G., Zhang S., Zentgraf T. (2017). Nonlinear photonic metasurfaces. *Nat. Rev. Mater.*.

[27] Grinblat G. (2021). Nonlinear dielectric nanoantennas and metasurfaces: frequency conversion and wavefront control. *ACS Photonics*.

[28] Gigli C., Leo G. (2022). All-dielectric *χ* (2) metasurfaces: recent progress. *Opto-Electron. Adv.*.

[29] Minovich A. E., Miroshnichenko A. E., Bykov A. Y., Murzina T. V., Neshev D. N., Kivshar Y. S. (2015). Functional and nonlinear optical metasurfaces. *Laser Photonics Rev.*.

[30] Sain B., Meier C., Zentgraf T. (2019). Nonlinear optics in all-dielectric nanoantennas and metasurfaces: a review. *Adv. Photonics*.

[31] Chen S., Li G., Cheah K. W., Zentgraf T., Zhang S. (2018). Controlling the phase of optical nonlinearity with plasmonic metasurfaces. *Nanophotonics*.

[32] Rahmani M., Leo G., Brener I. (2018). Nonlinear frequency conversion in optical nanoantennas and metasurfaces: materials evolution and fabrication. *Opto-Electron. Adv.*.

[33] Pertsch T., Kivshar Y. (2020). Nonlinear optics with resonant metasurfaces. *MRS Bull.*.

[34] Krasnok A., Tymchenko M., Alù A. (2018). Nonlinear metasurfaces: a paradigm shift in nonlinear optics. *Mater. Today*.

[35] Kolkowski R., Hakala T. K., Shevchenko A., Huttunen M. J. (2023). Nonlinear nonlocal metasurfaces. *Appl. Phys. Lett.*.

[36] Vabishchevich P., Kivshar Y. (2023). Nonlinear photonics with metasurfaces. *Photonics Res.*.

[37] Jeon D., Shin K., Moon S.-W., Rho J. (2023). Recent advancements of metalenses for functional imaging. *Nano Convergence*.

[38] Gennaro S., Sarma R., Brener I. (2022). Nonlinear and ultrafast all-dielectric metasurfaces at the center for integrated nanotechnologies. *Nanotechnology*.

[39] Keren-Zur S., Avayu O., Michaeli L., Ellenbogen T. (2016). Nonlinear beam shaping with plasmonic metasurfaces. *ACS Photonics*.

[40] Keren-Zur S., Michaeli L., Suchowski H., Ellenbogen T. (2018). Shaping light with nonlinear metasurfaces. *Adv. Opt. Photon*.

[41] Zhao Y., Yang Y., Sun H.-B. (2021). Nonlinear meta-optics towards applications. *PhotoniX*.

[42] Yuan S., Wu Y., Dang Z. (2021). Strongly enhanced second harmonic generation in a thin film lithium niobate heterostructure cavity. *Phys. Rev. Lett.*.

[43] Grinblat G., Li Y., Nielsen M. P., Oulton R. F., Maier S. A. (2016). Enhanced third harmonic generation in single germanium nanodisks excited at the anapole mode. *Nano Lett.*.

[44] Celebrano M., Rocco D., Gandolfi M. (2021). Optical tuning of dielectric nanoantennas for thermo-optically reconfigurable nonlinear metasurfaces. *Opt. Lett.*.

[45] Carletti L., Zilli A., Moia F. (2021). Steering and encoding the polarization of the second harmonic in the visible with a monolithic linbo3 metasurface. *ACS Photonics*.

[46] Löchner F. J. F., Fedotova A. N., Liu S. (2018). Polarization-dependent second harmonic diffraction from resonant gaas metasurfaces. *ACS Photonics*.

[47] Vabishchevich P. P., Liu S., Sinclair M. B., Keeler G. A., Peake G. M., Brener I. (2018). Enhanced second-harmonic generation using broken symmetry iii–v semiconductor fano metasurfaces. *ACS Photonics*.

[48] Liu S., Sinclair M. B., Saravi S. (2016). Resonantly enhanced second-harmonic generation using iii-v semiconductor all-dielectric metasurfaces. *Nano Lett.*.

[49] Okhlopkov K. I., Zilli A., Tognazzi A. (2021). Tailoring third-harmonic diffraction efficiency by hybrid modes in high-Q metasurfaces. *Nano Lett.*.

[50] Gandolfi M., Tognazzi A., Rocco D., De Angelis C., Carletti L. (2021). Near-unity third-harmonic circular dichroism driven by a quasibound state in the continuum in asymmetric silicon metasurfaces. *Phys. Rev. A*.

[51] Xu L., Smirnova D. A., Camacho-Morales R. (2022). Enhanced four-wave mixing from multi-resonant silicon dimer-hole membrane metasurfaces. *New J. Phys.*.

[52] Carletti L., Kruk S. S., Bogdanov A. A., De Angelis C., Kivshar Y. (2019). High-harmonic generation at the nanoscale boosted by bound states in the continuum. *Phys. Rev. Res.*.

[53] Zograf G., Koshelev K., Zalogina A. (2022). High-harmonic generation from resonant dielectric metasurfaces empowered by bound states in the continuum. *ACS Photonics*.

[54] Aouani H., Rahmani M., Navarro-Cía M., Maier S. A. (2014). Third-harmonic-upconversion enhancement from a single semiconductor nanoparticle coupled to a plasmonic antenna. *Nat. Nanotechnol.*.

[55] Anthur A. P., Zhang H., Paniagua-Dominguez R. (2020). Continuous wave second harmonic generation enabled by quasi-bound-states in the continuum on gallium phosphide metasurfaces. *Nano Lett.*.

[56] Tseng M. L., Semmlinger M., Zhang M. (2022). Vacuum ultraviolet nonlinear metalens. *Sci. Adv.*.

[57] Hsu C. W., Zhen B., Stone A. D., Joannopoulos J. D., Soljačić M. (2016). Bound states in the continuum. *Nat. Rev. Mater.*.

[58] Bernhardt N., Koshelev K., White S. J. (2020). Quasi-bic resonant enhancement of second-harmonic generation in ws2 monolayers. *Nano Lett.*.

[59] Xu L., Zangeneh Kamali K., Huang L. (2019). Dynamic nonlinear image tuning through magnetic dipole quasi-bic ultrathin resonators. *Adv. Sci.*.

[60] Schlickriede C., Waterman N., Reineke B. (2018). Imaging through nonlinear metalens using second harmonic generation. *Adv. Mater.*.

[61] del Rocio Camacho-Morales M., Rocco D., Xu L. (2021). Infrared upconversion imaging in nonlinear metasurfaces. *Adv. Photonics*.

[62] Zheng Z., Lei X., Huang L. (2023). Third-harmonic generation and imaging with resonant si membrane metasurface. *Opto-Electron. Adv.*.

[63] Zhou J., Zhao J., Wu Q. (2022). Nonlinear computational edge detection metalens. *Adv. Funct. Mater.*.

[64] Reineke B., Sain B., Zhao R. (2019). Silicon metasurfaces for third harmonic geometric phase manipulation and multiplexed holography. *Nano Lett.*.

[65] Liu B., Sain B., Reineke B. (2020). Nonlinear wavefront control by geometric-phase dielectric metasurfaces: influence of mode field and rotational symmetry. *Adv. Opt. Mater.*.

[66] Mao N., Tang Y., Jin M. (2021). Nonlinear wavefront engineering with metasurface decorated quartz crystal. *Nanophotonics*.

[67] Zilli A., Rocco D., Finazzi M. (2021). Frequency tripling via sum-frequency generation at the nanoscale. *ACS Photonics*.

[68] Tseng M. L., Jahani Y., Leitis A., Altug H. (2020). Dielectric metasurfaces enabling advanced optical biosensors. *ACS Photonics*.

[69] Sarovar M., Ishizaki A., Fleming G. R., Whaley K. B. (2010). Quantum entanglement in photosynthetic light-harvesting complexes. *Nat. Phys.*.

[70] Zhong H.-S., Wang H., Deng Y.-H. (2020). Quantum computational advantage using photons. *Science*.

[71] O’brien J. L. (2007). Optical quantum computing. *Science*.

[72] Solntsev A. S., Agarwal G. S., Kivshar Y. S. (2021). Metasurfaces for quantum photonics. *Nat. Photonics*.

[73] Marino G., Solntsev A. S., Xu L. (2019). Spontaneous photon-pair generation from a dielectric nanoantenna. *Optica*.

[74] Zhang J., Ma J., Parry M. (2022). Spatially entangled photon pairs from lithium niobate nonlocal metasurfaces. *Sci. Adv.*.

[75] Santiago-Cruz T., Gennaro S. D., Mitrofanov O. (2022). Resonant metasurfaces for generating complex quantum states. *Science*.

[76] Stav T., Faerman A., Maguid E. (2018). Quantum entanglement of the spin and orbital angular momentum of photons using metamaterials. *Science*.

[77] Jha P. K., Shitrit N., Kim J., Ren X., Wang Y., Zhang X. (2017). Metasurface-mediated quantum entanglement. *ACS Photonics*.

[78] Wang K., Titchener J. G., Kruk S. S. (2018). Quantum metasurface for multiphoton interference and state reconstruction. *Science*.

[79] Sultanov V., Santiago-Cruz T., Chekhova M. V. (2022). Flat-optics generation of broadband photon pairs with tunable polarization entanglement. *Opt. Lett.*.

[80] Butet J., Russier-Antoine I., Jonin C., Lascoux N., Benichou E., Brevet P.-F. (2012). Sensing with multipolar second harmonic generation from spherical metallic nanoparticles. *Nano Lett.*.

[81] Qin J., Jiang S., Wang Z. (2022). Metasurface micro/nano-optical sensors: principles and applications. *ACS Nano*.

[82] Gigli C., Marino G., Artioli A. (2021). Tensorial phase control in nonlinear meta-optics. *Optica*.

[83] Bloembergen N. (1996). *Nonlinear Optics*.

[84] Kulkarni G., Rioux J., Braverman B., Chekhova M. V., Boyd R. W. (2022). Classical model of spontaneous parametric down-conversion. *Phys. Rev. Res.*.

[85] Bloembergen N., Pershan P. S. (1962). Light waves at the boundary of nonlinear media. *Phys. Rev.*.

[86] Guyot-Sionnest P., Chen W., Shen Y. R. (1986). General considerations on optical second-harmonic generation from surfaces and interfaces. *Phys. Rev. B*.

[87] Sipe J. E., Moss D. J., van Driel H. M. (1987). Phenomenological theory of optical second- and third-harmonic generation from cubic centrosymmetric crystals. *Phys. Rev. B*.

[88] Heinz T., Ponath H.-E., Stegeman G. (1991). Chapter 5 – second-order nonlinear optical effects at surfaces and interfaces. *Nonlinear Surface Electromagnetic Phenomena, ser. Modern Problems in Condensed Matter Sciences*.

[89] de Beer A. G. F., Campen R. K., Roke S. (2010). Separating surface structure and surface charge with second-harmonic and sum-frequency scattering. *Phys. Rev. B*.

[90] Timbrell D., You J. W., Kivshar Y. S., Panoiu N. C. (2018). A comparative analysis of surface and bulk contributions to second-harmonic generation in centrosymmetric nanoparticles. *Sci. Rep.*.

[91] Panoiu N. C., Sha W. E., Lei D., Li G. (2018). Nonlinear optics in plasmonic nanostructures. *J. Opt.*.

[92] Smirnova D., Kivshar Y. S. (2016). Multipolar nonlinear nanophotonics. *Optica*.

[93] Bonacina L., Brevet P.-F., Finazzi M., Celebrano M. (2020). Harmonic generation at the nanoscale. *J. Appl. Phys.*.

[94] Liu T., Xiao S., Li B., Gu M., Luan H., Fang X. (2022). Third-and second-harmonic generation in all-dielectric nanostructures: a mini review. *Front. Nanotechnol.*.

[95] Armstrong J. A., Bloembergen N., Ducuing J., Pershan P. S. (1962). Interactions between light waves in a nonlinear dielectric. *Phys. Rev.*.

[96] Franken P. A., Ward J. F. (1963). Optical harmonics and nonlinear phenomena. *Rev. Mod. Phys.*.

[97] Bass M., Franken P. A., Hill A. E., Peters C. W., Weinreich G. (1962). Optical mixing. *Phys. Rev. Lett.*.

[98] Louisell W. H., Yariv A., Siegman A. E. (1961). Quantum fluctuations and noise in parametric processes. i. *Phys. Rev.*.

[99] Klyshko D. N. (1967). Coherent photon decay in a nonlinear medium. *JETP Lett*..

[100] Harris S. E., Oshman M. K., Byer R. L. (1967). Observation of tunable optical parametric fluorescence. *Phys. Rev. Lett.*.

[101] Akhmanov S. A., Fadeev V. V., Khokhlov R. V., Chunaev O. N. (1967). Quantum noise in parametric light amplifiers. *JETP Lett.*.

[102] Burnham D. C., Weinberg D. L. (1970). Observation of simultaneity in parametric production of optical photon pairs. *Phys. Rev. Lett.*.

[103] Midwinter J. (1968). Parametric infrared image converters. *IEEE J. Quantum Electron.*.

[104] Firester A. H. (1969). Parametric image conversion: Part i. *J. Appl. Phys.*.

[105] Firester A. H. (1969). Holography and parametric image conversion: Part II. *J. Appl. Phys.*.

[106] Terhune R. W., Maker P. D., Savage C. M. (1962). Optical harmonic generation in calcite. *Phys. Rev. Lett.*.

[107] Kleinman D. A. (1962). Nonlinear dielectric polarization in optical media. *Phys. Rev.*.

[108] Jha S. S., Bloembergen N. (1968). Nonlinear optical susceptibilities in group-iv and iii-v semiconductors. *Phys. Rev.*.

[109] Burns W. K., Bloembergen N. (1971). Third-harmonic generation in absorbing media of cubic or isotropic symmetry. *Phys. Rev. B*.

[110] Aspnes D. E. (1972). Energy-band theory of the second-order nonlinear optical susceptibility of crystals of zinc-blende symmetry. *Phys. Rev. B*.

[111] Dadap J. I., Shan J., Eisenthal K. B., Heinz T. F. (1999). Second-harmonic Rayleigh scattering from a sphere of centrosymmetric material. *Phys. Rev. Lett.*.

[112] Dadap J. I., Shan J., Heinz T. F. (2004). Theory of optical second-harmonic generation from a sphere of centrosymmetric material: small-particle limit. *J. Opt. Soc. Am. B*.

[113] de Beer A. G. F., Roke S. (2007). Sum frequency generation scattering from the interface of an isotropic particle: geometrical and chiral effects. *Phys. Rev. B*.

[114] Finazzi M., Biagioni P., Celebrano M., Duò L. (2007). Selection rules for second-harmonic generation in nanoparticles. *Phys. Rev. B*.

[115] de Beer A. G. F., Roke S., Dadap J. I. (2011). Theory of optical second-harmonic and sum-frequency scattering from arbitrarily shaped particles. *J. Opt. Soc. Am. B*.

[116] Kolkowski R., Szeszko J., Dwir B., Kapon E., Zyss J. (2016). Non-centrosymmetric plasmonic crystals for second-harmonic generation with controlled anisotropy and enhancement. *Laser Photonics Rev.*.

[117] Kapshai V. (2018). Sum-frequency generation from a thin cylindrical layer. *Opt. Spectrosc.*.

[118] Celebrano M., Locatelli A., Ghirardini L. (2019). Evidence of cascaded third-harmonic generation in noncentrosymmetric gold nanoantennas. *Nano Lett.*.

[119] Lovesey S. W., van der Laan G. (2019). Circular dichroism of second harmonic generation response. *Phys. Rev. B*.

[120] Mandujano M. A. G., Méndez E. R., Valencia C. I., Mendoza B. S. (2019). Multipolar analysis of the second harmonic generated by dielectric particles. *Opt. Express*.

[121] Frizyuk K. (2019). Second-harmonic generation in dielectric nanoparticles with different symmetries. *J. Opt. Soc. Am. B*.

[122] Frizyuk K., Volkovskaya I., Smirnova D., Poddubny A., Petrov M. (2019). Second-harmonic generation in mie-resonant dielectric nanoparticles made of noncentrosymmetric materials. *Phys. Rev. B*.

[123] Frizyuk K., Melik-Gaykazyan E., Choi J.-H., Petrov M. I., Park H.-G., Kivshar Y. (2021). Nonlinear circular dichroism in mie-resonant nanoparticle dimers. *Nano Lett.*.

[124] Nikitina A., Nikolaeva A., Frizyuk K. (2023). Nonlinear circular dichroism in achiral dielectric nanoparticles. *Phys. Rev. B*.

[125] Celebrano M., Wu X., Baselli M. (2015). Mode matching in multiresonant plasmonic nanoantennas for enhanced second harmonic generation. *Nat. Nanotechnol.*.

[126] Lee J., Tymchenko M., Argyropoulos C. (2014). Giant nonlinear response from plasmonic metasurfaces coupled to intersubband transitions. *Nature*.

[127] Liberal I., Engheta N. (2017). Near-zero refractive index photonics. *Nat. Photonics*.

[128] Reshef O., De Leon I., Alam M. Z., Boyd R. W. (2019). Nonlinear optical effects in epsilon-near-zero media. *Nat. Rev. Mater.*.

[129] Rocco D., De Angelis C., De Ceglia D., Carletti L., ichael, Vincenti M. A. (2020). Dielectric nanoantennas on epsilon-near-zero substrates: impact of losses on second order nonlinear processes. *Opt. Commun.*.

[130] Deng J., Tang Y., Chen S., Li K., Zayats A. V., Li G. (2020). Giant enhancement of second-order nonlinearity of epsilon-near-zero medium by a plasmonic metasurface. *Nano Lett.*.

[131] Huang L., Xu L., Powell D. A., Padilla W. J., Miroshnichenko A. E. (2023). Resonant leaky modes in all-dielectric metasystems: fundamentals and applications. *Phys. Rep.*.

[132] Yang Y., Wang W., Boulesbaa A. (2015). Nonlinear fano-resonant dielectric metasurfaces. *Nano Lett.*.

[133] Shorokhov A. S., Melik-Gaykazyan E. V., Smirnova D. A. (2016). Multifold enhancement of third-harmonic generation in dielectric nanoparticles driven by magnetic fano resonances. *Nano Lett.*.

[134] Chen S., Rahmani M., Li K. F. (2018). Third harmonic generation enhanced by multipolar interference in complementary silicon metasurfaces. *ACS Photonics*.

[135] Semmlinger M., Zhang M., Tseng M. L. (2019). Generating third harmonic vacuum ultraviolet light with a tio2 metasurface. *Nano Lett.*.

[136] Smirnova D., Kruk S., Leykam D., Melik-Gaykazyan E., Choi D.-Y., Kivshar Y. (2019). Third-harmonic generation in photonic topological metasurfaces. *Phys. Rev. Lett.*.

[137] Fedotova A., Younesi M., Sautter J. (2020). Second-harmonic generation in resonant nonlinear metasurfaces based on lithium niobate. *Nano Lett.*.

[138] Liu Z., Xu Y., Lin Y. (2019). High-q quasibound states in the continuum for nonlinear metasurfaces. *Phys. Rev. Lett.*.

[139] Koshelev K., Tang Y., Li K., Choi D.-Y., Li G., Kivshar Y. (2019). Nonlinear metasurfaces governed by bound states in the continuum. *ACS Photonics*.

[140] Yang G., Dev S. U., Allen M. S., Allen J. W., Harutyunyan H. (2022). Optical bound states in the continuum enabled by magnetic resonances coupled to a mirror. *Nano Lett.*.

[141] Camacho-Morales R., Xu L., Zhang H. (2022). Sum-frequency generation in high-q gap metasurfaces driven by leaky-wave guided modes. *Nano Lett.*.

[142] Hong P., Xu L., Rahmani M. (2022). Dual bound states in the continuum enhanced second harmonic generation with transition metal dichalcogenides monolayer. *Opto-Electron. Adv.*.

[143] Tilmann B., Grinblat G., Berté R. (2020). Nanostructured amorphous gallium phosphide on silica for nonlinear and ultrafast nanophotonics. *Nanoscale Horiz.*.

[144] Marino G., Rocco D., Gigli C. (2021). Harmonic generation with multi-layer dielectric metasurfaces. *Nanophotonics*.

[145] Marino G., Gigli C., Rocco D. (2019). Zero-order second harmonic generation from algaas-on-insulator metasurfaces. *ACS Photonics*.

[146] Semmlinger M., Tseng M. L., Yang J. (2018). Vacuum ultraviolet light-generating metasurface. *Nano Lett.*.

[147] Carletti L., Li C., Sautter J. (2019). Second harmonic generation in monolithic lithium niobate metasurfaces. *Opt. Express*.

[148] Ma J., Xie F., Chen W. (2021). Nonlinear lithium niobate metasurfaces for second harmonic generation. *Laser Photonics Rev.*.

[149] Liu T., Qin M., Wu F., Xiao S. (2023). High-efficiency optical frequency mixing in an all-dielectric metasurface enabled by multiple bound states in the continuum. *Phys. Rev. B*.

[150] Shafirin P. A., Zubyuk V. V., Fedyanin A. A., Shcherbakov M. R. (2022). Nonlinear response of q-boosting metasurfaces beyond the time-bandwidth limit. *Nanophotonics*.

[151] Tonkaev P., Koshelev K., Masharin M. A., Makarov S. V., Kruk S. S., Kivshar Y. (2023). Observation of enhanced generation of a fifth harmonic from halide perovskite nonlocal metasurfaces. *ACS Photonics*.

[152] Liu S., Vabishchevich P. P., Vaskin A. (2018). An all-dielectric metasurface as a broadband optical frequency mixer. *Nat. Commun.*.

[153] Zheng Z., Xu L., Huang L. (2022). Boosting second-harmonic generation in the linbo 3 metasurface using high-q guided resonances and bound states in the continuum. *Phys. Rev. B*.

[154] Zhu M., Abdollahramezani S., Fan T., Adibi A. (2021). Dynamically tunable third-harmonic generation with all-dielectric metasurfaces incorporating phase-change chalcogenides. *Opt. Lett.*.

[155] Abdelraouf O. A., Anthur A. P., Dong Z. (2021). Multistate tuning of third harmonic generation in fano-resonant hybrid dielectric metasurfaces. *Adv. Funct. Mater.*.

[156] Gao J., Vincenti M. A., Frantz J. (2021). Near-infrared to ultra-violet frequency conversion in chalcogenide metasurfaces. *Nat. Commun.*.

[157] Smirnova D. A., Khanikaev A. B., Smirnov L. A., Kivshar Y. S. (2016). Multipolar third-harmonic generation driven by optically induced magnetic resonances. *ACS Photonics*.

[158] Wang L., Kruk S., Koshelev K., Kravchenko I., Luther-Davies B., Kivshar Y. (2018). Nonlinear wavefront control with all-dielectric metasurfaces. *Nano Lett.*.

[159] Volkovskaya I., Xu L., Huang L., Smirnov A. I., Miroshnichenko A. E., Smirnova D. (2020). Multipolar second-harmonic generation from high-q quasi-bic states in subwavelength resonators. *Nanophotonics*.

[160] Xu L., Saerens G., Timofeeva M. (2019). Forward and backward switching of nonlinear unidirectional emission from gaas nanoantennas. *ACS Nano*.

[161] Rocco D., Zilli A., Ferraro A. (2022). Tunable second harmonic generation by an all-dielectric diffractive metasurface embedded in liquid crystals. *New J. Phys.*.

[162] Leon U. A., Rocco D., Carletti L. (2022). Thz-photonics transceivers by all-dielectric phonon-polariton nonlinear nanoantennas. *Sci. Rep.*.

[163] Gandhi H. K., Rocco D., Carletti L., De Angelis C. (2020). Gain-loss engineering of bound states in the continuum for enhanced nonlinear response in dielectric nanocavities. *Opt. Express*.

[164] Huang L., Zhang S., Zentgraf T. (2018). Metasurface holography: from fundamentals to applications. *Nanophotonics*.

[165] Zheng G., Mühlenbernd H., Kenney M., Li G., Zentgraf T., Zhang S. (2015). Metasurface holograms reaching 80% efficiency. *Nat. Nanotechnol.*.

[166] Gao H., Fan X., Xiong W., Hong M. (2021). Recent advances in optical dynamic meta-holography. *Opto-Electron. Adv.*.

[167] Walter F., Li G., Meier C., Zhang S., Zentgraf T. (2017). Ultrathin nonlinear metasurface for optical image encoding. *Nano Lett.*.

[168] Tang Y., Intaravanne Y., Deng J., Li K. F., Chen X., Li G. (2019). Nonlinear vectorial metasurface for optical encryption. *Phys. Rev. Appl.*.

[169] Mao N., Deng J., Zhang X. (2020). Nonlinear diatomic metasurface for real and fourier space image encoding. *Nano Lett.*.

[170] Almeida E., Bitton O., Prior Y. (2016). Nonlinear metamaterials for holography. *Nat. Commun.*.

[171] Gao Y., Fan Y., Wang Y., Yang W., Song Q., Xiao S. (2018). Nonlinear holographic all-dielectric metasurfaces. *Nano Lett.*.

[172] Ye W., Zeuner F., Li X. (2016). Spin and wavelength multiplexed nonlinear metasurface holography. *Nat. Commun.*.

[173] Zhao W., Wang K., Hong X. (2020). Chirality-selected second-harmonic holography with phase and binary amplitude manipulation. *Nanoscale*.

[174] Ghirardini L., Marino G., Gili V. F. (2018). Shaping the nonlinear emission pattern of a dielectric nanoantenna by integrated holographic gratings. *Nano Lett.*.

[175] Lin Z., Huang L., Xu Z. T., Li X., Zentgraf T., Wang Y. (2019). Four-wave mixing holographic multiplexing based on nonlinear metasurfaces. *Adv. Opt. Mater.*.

[176] Fang X., Yang H., Yao W. (2021). High-dimensional orbital angular momentum multiplexing nonlinear holography. *Adv. Photonics*.

[177] Karnieli A., Li Y., Arie A. (2022). The geometric phase in nonlinear frequency conversion. *Front. Phys.*.

[178] Gao Z., Genevet P., Li G., Dorfman K. E. (2021). Reconstruction of multidimensional nonlinear polarization response of pancharatnam-berry metasurfaces. *Phys. Rev. B*.

[179] Hong X., Wang K., Guan C. (2022). Chiral third-harmonic metasurface for multiplexed holograms. *Nano Lett.*.

[180] Li G., Sartorello G., Chen S. (2018). Spin and geometric phase control four-wave mixing from metasurfaces. *Laser Photonics Rev.*.

[181] Almeida E., Shalem G., Prior Y. (2016). Subwavelength nonlinear phase control and anomalous phase matching in plasmonic metasurfaces. *Nat. Commun.*.

[182] Blechman Y., Tsesses S., Bartal G., Almeida E. (2022). Inverse design of broadband, strongly-coupled plexcitonic nonlinear metasurfaces. *New J. Phys.*.

[183] Kruk S. S., Wang L., Sain B. (2022). Asymmetric parametric generation of images with nonlinear dielectric metasurfaces. *Nat. Photonics*.

[184] Ma M., Li Z., Liu W. (2019). Optical information multiplexing with nonlinear coding metasurfaces. *Laser Photonics Rev.*.

[185] Schlickriede C., Kruk S. S., Wang L., Sain B., Kivshar Y., Zentgraf T. (2020). Nonlinear imaging with all-dielectric metasurfaces. *Nano Lett.*.

[186] Rocco D., Gigli C., Carletti L. (2020). Vertical second harmonic generation in asymmetric dielectric nanoantennas. *IEEE Photonics J.*.

[187] Ossiander M., Meretska M. L., Hampel H. K. (2023). Extreme ultraviolet metalens by vacuum guiding. *Science*.

[188] Ahmadivand A., Semmlinger M., Dong L., Gerislioglu B., Nordlander P., Halas N. J. (2019). Toroidal dipole-enhanced third harmonic generation of deep ultraviolet light using plasmonic meta-atoms. *Nano Lett.*.

[189] Timpu F., Reig Escalé M., Timofeeva M. (2019). Enhanced nonlinear yield from barium titanate metasurface down to the near ultraviolet. *Adv. Opt. Mater.*.

[190] Gao J., Vincenti M. A., Frantz J. (2022). All-optical tunable wavelength conversion in opaque nonlinear nanostructures. *Nanophotonics*.

[191] Nikolaeva A., Mastalieva V., Gudovskikh A. S. (2022). Large-scale flexible membrane with resonant silicon nanowires for infrared visualization via efficient third harmonic generation. *Appl. Phys. Lett.*.

[192] Saerens G., Bloch E., Frizyuk K. (2022). Second-harmonic generation tuning by stretching arrays of gaas nanowires. *Nanoscale*.

[193] Fedorov V. V., Bolshakov A., Sergaeva O. (2020). Gallium phosphide nanowires in a free-standing, flexible, and semitransparent membrane for large-scale infrared-to-visible light conversion. *ACS Nano*.

[194] De Ceglia D., De Angelis C. (2023). Image processing with nonlocal nonlinear metasurfaces. *The 44th PIERS 2023 Conference in Prague*.

[195] Garay-Palmett K., Kim D. B., Zhang Y., Domínguez-Serna F. A., Lorenz V. O., U’Ren A. B. (2023). Fiber-based photon-pair generation: tutorial. *JOSA B*.

[196] Cohen O., Lundeen J. S., Smith B. J., Puentes G., Mosley P. J., Walmsley I. A. (2009). Tailored photon-pair generation in optical fibers. *Phys. Rev. Lett.*.

[197] Smith B. J., Mahou P., Cohen O., Lundeen J., Walmsley I. (2009). Photon pair generation in birefringent optical fibers. *Opt. Express*.

[198] Li X., Chen J., Voss P., Sharping J., Kumar P. (2004). All-fiber photon-pair source for quantum communications: improved generation of correlated photons. *Opt. Express*.

[199] Helt L., Steel M., Sipe J. (2015). Spontaneous parametric downconversion in waveguides: what’s loss got to do with it?. *New J. Phys.*.

[200] Ngo G. Q., Najafidehaghani E., Gan Z. (2022). In-fibre second-harmonic generation with embedded two-dimensional materials. *Nat. Photonics*.

[201] Vlasov Y. A., McNab S. J. (2004). Losses in single-mode silicon-on-insulator strip waveguides and bends. *Opt. Express*.

[202] Melati D., Melloni A., Morichetti F. (2014). Real photonic waveguides: guiding light through imperfections. *Adv. Opt. Photonics*.

[203] Kuznetsov A. I., Miroshnichenko A. E., Brongersma M. L., Kivshar Y. S., Luk’yanchuk B. (2016). Optically resonant dielectric nanostructures. *Science*.

[204] Santiago-Cruz T., Fedotova A., Sultanov V. (2021). Photon pairs from resonant metasurfaces. *Nano Lett.*.

[205] Son C., Sultanov V., Santiago-Cruz T. (2023). Photon pairs bi-directionally emitted from a resonant metasurface. *Nanoscale*.

[206] Poddubny A. N., Iorsh I. V., Sukhorukov A. A. (2016). Generation of photon-plasmon quantum states in nonlinear hyperbolic metamaterials. *Phys. Rev. Lett.*.

[207] Lenzini F., Poddubny A. N., Titchener J. (2018). Direct characterization of a nonlinear photonic circuit’s wave function with laser light. *Light: Sci. Appl.*.

[208] Jin B., Mishra D., Argyropoulos C. (2021). Efficient single-photon pair generation by spontaneous parametric down-conversion in nonlinear plasmonic metasurfaces. *Nanoscale*.

[209] Parry M., Mazzanti A., Poddubny A., Valle G. D., Neshev D. N., Sukhorukov A. A. (2021). Enhanced generation of nondegenerate photon pairs in nonlinear metasurfaces. *Adv. Photonics*.

[210] Nielsen M. A., Chuang I. (2010). *Quantum Computation and Quantum Information*.

[211] Ekert A. K. (1991). Quantum cryptography based on bell’s theorem. *Phys. Rev. Lett.*.

[212] Gottesman D. (1998). *The Heisenberg Representation of Quantum Computers*.

[213] Hein M., Eisert J., Briegel H. J. (2004). Multiparty entanglement in graph states. *Phys. Rev. A*.

[214] Gross D., Liu Y.-K., Flammia S. T., Becker S., Eisert J. (2010). Quantum state tomography via compressed sensing. *Phys. Rev. Lett.*.

[215] Carolan J., Harrold C., Sparrow C. (2015). Universal linear optics. *Science*.

[216] Orieux A., Versteegh M. A., Jöns K. D., Ducci S. (2017). Semiconductor devices for entangled photon pair generation: a review. *Rep. Prog. Phys.*.

[217] Müller M., Bounouar S., Jöns K. D., Glässl M., Michler P. (2014). On-demand generation of indistinguishable polarization-entangled photon pairs. *Nat. Photonics*.

[218] Weston M. M., Chrzanowski H. M., Wollmann S. (2016). Efficient and pure femtosecond-pulse-length source of polarization-entangled photons. *Opt. Express*.

[219] Barbieri M., Cinelli C., Mataloni P., De Martini F. (2005). Polarization-momentum hyperentangled states: realization and characterization. *Phys. Rev. A*.

[220] Brendel J., Gisin N., Tittel W., Zbinden H. (1999). Pulsed energy-time entangled twin-photon source for quantum communication. *Phys. Rev. Lett.*.

[221] Franson J. D. (1989). Bell inequality for position and time. *Phys. Rev. Lett.*.

[222] Kok P., Munro W. J., Nemoto K., Ralph T. C., Dowling J. P., Milburn G. J. (2007). Linear optical quantum computing with photonic qubits. *Rev. Mod. Phys.*.

[223] Gisin N., Thew R. (2007). Quantum communication. *Nat. Photonics*.

[224] Weissflog M. A., Dezert R., Vinel V. (2023). Nonlinear nanoresonators are natural sources of bell states. ..

[225] Gao Y.-J., Wang Z., Jiang Y. (2022). Multichannel distribution and transformation of entangled photons with dielectric metasurfaces. *Phys. Rev. Lett.*.

[226] Zhang D., Chen Y., Gong S. (2022). All-optical modulation of quantum states by nonlinear metasurface. *Light: Sci. Appl.*.

[227] Yu J., Park S., Hwang I. (2022). Electrically tunable nonlinear polaritonic metasurface. *Nat. Photonics*.

[228] Kim Y., Wu P. C., Sokhoyan R. (2019). Phase modulation with electrically tunable vanadium dioxide phase-change metasurfaces. *Nano Lett.*.

[229] Ren M.-X., Wu W., Cai W., Pi B., Zhang X.-Z., Xu J.-J. (2017). Reconfigurable metasurfaces that enable light polarization control by light. *Light: Sci. Appl.*.

[230] Pedersen S. P., Zhang L., Pohl T. (2023). Quantum nonlinear metasurfaces from dual arrays of ultracold atoms. *Phys. Rev. Res.*.

[231] Zhang X. G., Sun Y. L., Zhu B. (2022). A metasurface-based light-to-microwave transmitter for hybrid wireless communications. *Light: Sci. Appl.*.

[232] Ðorđević N., Schwanninger R., Yarema M. (2022). Metasurface colloidal quantum dot photodetectors. *ACS Photonics*.

[233] Bao Y., Lin Q., Su R. (2020). On-demand spin-state manipulation of single-photon emission from quantum dot integrated with metasurface. *Sci. Adv.*.

[234] Hsu T.-W., Zhu W., Thiele T. (2022). Single-atom trapping in a metasurface-lens optical tweezer. *PRX Quantum*.

[235] Zeng T.-Y., Liu G.-D., Wang L.-L., Lin Q. (2021). Light-matter interactions enhanced by quasi-bound states in the continuum in a graphene-dielectric metasurface. *Opt. Express*.

[236] Li Z.-X., Zhu D., Lin P.-C. (2022). High-dimensional entanglement generation based on a pancharatnam–berry phase metasurface. *Photonics Res.*.

[237] Titchener J. G., Gräfe M., Heilmann R., Solntsev A. S., Szameit A., Sukhorukov A. A. (2018). Scalable on-chip quantum state tomography. *Npj Quantum Inf.*.

[238] Foreman M. R., Favaro A., Aiello A. (2015). Optimal frames for polarization state reconstruction. *Phys. Rev. Lett.*.

[239] Zheng J., Xiao Y., Hu M. (2022). A single-photon-sensitivity spectrometer based on metasurfaces. *CLEO: QELS_Fundamental Science*.

[240] Georgi P., Massaro M., Luo K.-H. (2019). Metasurface interferometry toward quantum sensors. *Light: Sci. Appl.*.

[241] Zhang S., Wong C. L., Zeng S. (2020). Metasurfaces for biomedical applications: imaging and sensing from a nanophotonics perspective. *Nanophotonics*.

[242] Li Q., Orcutt K., Cook R. L. (2023). Single-photon absorption and emission from a natural photosynthetic complex. *Nature*.

[243] Khan S. A., Khan N. Z., Xie Y. (2022). Optical sensing by metamaterials and metasurfaces: from physics to biomolecule detection. *Adv. Opt. Mater.*.

[244] Du W., Wen X., Gérard D., Qiu C.-W., Xiong Q. (2020). Chiral plasmonics and enhanced chiral light-matter interactions. *Sci. China: Phys. Mech. Astron.*.

[245] Solomon M. L., Hu J., Lawrence M., García-Etxarri A., Dionne J. A. (2018). Enantiospecific optical enhancement of chiral sensing and separation with dielectric metasurfaces. *ACS Photonics*.

[246] Chen Y., Zhao C., Zhang Y., Qiu C.-w. (2020). Integrated molar chiral sensing based on high-q metasurface. *Nano Lett.*.

[247] Garcia-Guirado J., Svedendahl M., Puigdollers J., Quidant R. (2019). Enhanced chiral sensing with dielectric nanoresonators. *Nano Lett.*.

[248] Juan M. L., Gordon R., Pang Y., Eftekhari F., Quidant R. (2009). Self-induced back-action optical trapping of dielectric nanoparticles. *Nat. Phys.*.

[249] Mestres P., Berthelot J., Aćimović S. S., Quidant R. (2016). Unraveling the optomechanical nature of plasmonic trapping. *Light: Sci. Appl.*.

[250] Pang Y., Gordon R. (2012). Optical trapping of a single protein. *Nano Lett.*.

[251] Wheaton S., Gelfand R. M., Gordon R. (2015). Probing the Raman-active acoustic vibrations of nanoparticles with extraordinary spectral resolution. *Nat. Photonics*.

[252] Yousefi A., Ying C., Parmenter C. D. (2023). Optical monitoring of in situ iron loading into single, native ferritin proteins. *Nano Lett.*.

[253] Babaei E., Wright D., Gordon R. (2023). Fringe dielectrophoresis nanoaperture optical trapping with order of magnitude speed-up for unmodified proteins. *Nano Lett.*.

[254] Ying C., Karakaci E., Bermudez-Urena E. (2021). Watching single unmodified enzymes at work. ..

[255] Raman C. V. (1928). A change of wave-length in light scattering. *Nature*.

[256] Gardiner D., Graves P., Bowley H. (1989). *Practical Raman Spectroscopy*.

[257] Fleischmann M., Hendra P. J., McQuillan A. J. (1974). Raman spectra of pyridine adsorbed at a silver electrode. *Chem. Phys. Lett.*.

[258] Gwo S., Wang C.-Y., Chen H.-Y. (2016). Plasmonic metasurfaces for nonlinear optics and quantitative sers. *ACS Photonics*.

[259] Palermo G., Rippa M., Conti Y. (2021). Plasmonic metasurfaces based on pyramidal nanoholes for high-efficiency sers biosensing. *ACS Appl. Mater. Interfaces*.

[260] Zeng Y., Ananth R., Dill T. J. (2022). Metasurface-enhanced Raman spectroscopy (msers) for oriented molecular sensing. *ACS Appl. Mater. Interfaces*.

[261] Mahigir A., Chang T.-W., Behnam A., Liu G. L., Gartia M. R., Veronis G. (2017). Plasmonic nanohole array for enhancing the sers signal of a single layer of graphene in water. *Sci. Rep.*.

[262] Huang J.-A., Mousavi M. Z., Zhao Y. (2019). Sers discrimination of single dna bases in single oligonucleotides by electro-plasmonic trapping. *Nat. Commun.*.

[263] Siddique R. H., Kumar S., Narasimhan V., Kwon H., Choo H. (2019). Aluminum metasurface with hybrid multipolar plasmons for 1000-fold broadband visible fluorescence enhancement and multiplexed biosensing. *ACS Nano*.

[264] Sugimoto H., Yashima S., Fujii M. (2018). Hybridized plasmonic gap mode of gold nanorod on mirror nanoantenna for spectrally tailored fluorescence enhancement. *ACS Photonics*.

[265] Anăstăsoaie V., Tomescu R., Kusko C. (2022). Influence of random plasmonic metasurfaces on fluorescence enhancement. *Materials*.

[266] Narasimhan V., Siddique R. H., Hoffmann M., Kumar S., Choo H. (2019). Enhanced broadband fluorescence detection of nucleic acids using multipolar gap-plasmons on biomimetic au metasurfaces. *Nanoscale*.

[267] Zhang W., Ding F., Li W.-D., Wang Y., Hu J., Chou S. Y. (2012). Giant and uniform fluorescence enhancement over large areas using plasmonic nanodots in 3d resonant cavity nanoantenna by nanoimprinting. *Nanotechnology*.

[268] Puchkova A., Vietz C., Pibiri E. (2015). Dna origami nanoantennas with over 5000-fold fluorescence enhancement and single-molecule detection at 25 *μ*m. *Nano Lett.*.

[269] Caldarola M., Albella P., Cortés E. (2015). Non-plasmonic nanoantennas for surface enhanced spectroscopies with ultra-low heat conversion. *Nat. Commun.*.

[270] Patel S. K., Parmar J., Kosta Y. P. (2020). Design of graphene metasurface based sensitive infrared biosensor. *Sens. Actuators, A*.

[271] Romano S., Zito G., Managò S. (2018). Surface-enhanced Raman and fluorescence spectroscopy with an all-dielectric metasurface. *J. Phys. Chem. C*.

[272] Staude I., Khardikov V. V., Fofang N. T. (2015). Shaping photoluminescence spectra with magnetoelectric resonances in all-dielectric nanoparticles. *ACS Photonics*.

[273] Regmi R., Berthelot J., Winkler P. M. (2016). All-dielectric silicon nanogap antennas to enhance the fluorescence of single molecules. *Nano Lett.*.

[274] Bouchet D., Mivelle M., Proust J. (2016). Enhancement and inhibition of spontaneous photon emission by resonant silicon nanoantennas. *Phys. Rev. Appl.*.

[275] Iwanaga M. (2020). All-dielectric metasurface fluorescence biosensors for high-sensitivity antibody/antigen detection. *ACS Nano*.

[276] Rissin D. M., Kan C. W., Campbell T. G. (2010). Single-molecule enzyme-linked immunosorbent assay detects serum proteins at subfemtomolar concentrations. *Nat. Biotechnol.*.

[277] Kim S. H., Iwai S., Araki S., Sakakihara S., Iino R., Noji H. (2012). Large-scale femtoliter droplet array for digital counting of single biomolecules. *Lab Chip*.

[278] Luan J., Seth A., Gupta R. (2020). Ultrabright fluorescent nanoscale labels for the femtomolar detection of analytes with standard bioassays. *Nat. Biomed. Eng.*.

[279] Iwanaga M. (2022). Rapid detection of attomolar sars-cov-2 nucleic acids in all-dielectric metasurface biosensors. *Biosensors*.

[280] Yi Z., Gaofeng L., Zhongquan W., Zhihai Z., Zhengguo S., Gang C. (2021). Recent research progress in optical super-resolution planar meta-lenses. *Opto-Electron. Eng.*.

[281] Conteduca D., Barth I., Pitruzzello G., Reardon C. P., Martins E. R., Krauss T. F. (2021). Dielectric nanohole array metasurface for high-resolution near-field sensing and imaging. *Nat. Commun.*.

[282] Tran R. J., Sly K. L., Conboy J. C. (2017). Applications of surface second harmonic generation in biological sensing. *Annu. Rev. Anal. Chem.*.

[283] Sahu S. P., Mahigir A., Chidester B., Veronis G., Gartia M. R. (2019). Ultrasensitive three-dimensional orientation imaging of single molecules on plasmonic nanohole arrays using second harmonic generation. *Nano Lett.*.

